# SC-AOF: A Sliding Camera and Asymmetric Optical-Flow-Based Blending Method for Image Stitching

**DOI:** 10.3390/s24134035

**Published:** 2024-06-21

**Authors:** Jiayi Chang, Qing Li, Yanju Liang, Liguo Zhou

**Affiliations:** 1 Institute of Microelectronics, Chinese Academy of Sciences, Beijing 100049, China; 2 University of Chinese Academy of Sciences, Beijing 101408, China; liqing@ime.ac.cn; 3 Wuxi Iot Innovation Center Co., Ltd., Wuxi 214135, China; liangyanju@wiot.tech; 4 School of Computation, Information and Technology, Technical University of Munich, 85748 Garching bei München, Germany

**Keywords:** image stitching, sliding cameras, asymmetric optical flow, image blending

## Abstract

Parallax processing and structure preservation have long been important and challenging tasks in image stitching. In this paper, an image stitching method based on sliding camera to eliminate perspective deformation and asymmetric optical flow to solve parallax is proposed. By maintaining the viewpoint of two input images in the mosaic non-overlapping area and creating a virtual camera by interpolation in the overlapping area, the viewpoint is gradually transformed from one to another so as to complete the smooth transition of the two image viewpoints and reduce perspective deformation. Two coarsely aligned warped images are generated with the help of a global projection plane. After that, the optical flow propagation and gradient descent method are used to quickly calculate the bidirectional asymmetric optical flow between the two warped images, and the optical-flow-based method is used to further align the two warped images to reduce parallax. In the image blending, the softmax function and registration error are used to adjust the width of the blending area, further eliminating ghosting and reducing parallax. Finally, by comparing our method with APAP, AANAP, SPHP, SPW, TFT, and REW, it has been proven that our method can not only effectively solve perspective deformation, but also gives more natural transitions between images. At the same time, our method can robustly reduce local misalignment in various scenarios, with higher structural similarity index. A scoring method combining subjective and objective evaluations of perspective deformation, local alignment and runtime is defined and used to rate all methods, where our method ranks first.

## 1. Introduction

Image stitching is a technology that can align and blend multiple images to generate a high-resolution, wide field-of-view and artifact-free mosaic. It has broad and promising applications in many fields such as virtual reality, remote sensing mapping, and urban modeling. The calculation of the global homography, as an important step in image stitching [1,2], directly determines the image alignment accuracy and the final user experience. However, global homography only works for planar scenes or rotation-only camera motions. For non-planar scenes or when the optical centers of cameras do not coincide, homography tends to cause misalignment, resulting in blurring and ghosting in the mosaic. It can also cause perspective deformation, making the final mosaic blurred and severely stretched at the edges. Many solutions have been proposed to solve the problems of parallax and perspective deformation in image stitching, so as to improve the quality of stitched images. But most state-of-the-art mesh-based [3,4,5] and multi-plane [6,7,8] methods are time-consuming and vulnerable to false matches. 

In this work, an innovative image stitching method combining sliding camera (SC) and asymmetric optical flow (AOF), referred to as the SC-AOF method, is proposed to reduce both perspective deformation and alignment error. In the non-overlapping area of the mosaic, the SC-AOF method manages to keep the viewpoint of the mosaic the same as or one rotation around the camera Z axis from those of the input images. In the overlapping area of the mosaic, the viewpoint is changed from one input image viewpoint to another, which can effectively solve the perspective deformation at the edge. A global projection plane is estimated to project input images onto the mosaic. After that, an asymmetric optical flow method is employed to further align the images. In the blending, the softmax function and alignment error are used to dynamically adjust the width of the blending area to further eliminate ghosting and improve the mosaic quality. This paper makes the following contributions:

- The SC-AOF method innovatively uses an approach based on sliding camera to reduce perspective deformation. Combined with either a global projection model or a local projection model, this method can effectively reduce the perspective deformation.
- An optical-flow-based image alignment and blending method is adopted to further mitigate misalignment and improve the stitching quality of the mosaic generated by a global projection model.
- Each step in the SC-AOF method can be combined with other methods to improve the stitching quality of those methods.

This article is organized as follows. Section 2 presents the related works. Section 3 first introduces the overall method of this article, then an edge stretching reduction method based on sliding camera and a local misalignment reduction method based on asymmetric optical flow are elaborated in detail. Section 4 presents our qualitative and quantitative experimental results compared with other methods. Finally, Section 5 summarizes our method.

## 2. Related Works

For the local alignment, APAP (as-projective-as-possible) [8,9] uses the weighted DLT (direct linear transform) method to estimate the location-dependent homography and then eliminate misalignment. However, if some key points match incorrectly, the image areas near these key points may have incorrect homography, resulting in serious alignment errors and distortion. APAP needs to estimate homography using DLT for each image cell, and therefore APAP runs much slower than the global homography warping. REW (robust elastic warping) [10,11] uses the TPS (thin-plate spline) interpolation method to convert discrete matched feature points into a deformation field, which is used to warp the image and achieve accurate local alignment. The estimation of TPS parameters and the deformation field is fast, so REW has excellent running efficiency. TFT (triangular facet approximation) [6] uses the Delaunay triangulation method and the matched feature points to triangulate the mosaic canvas, and the warping inside each triangle is determined by the homography calculated based on the three triangle vertices, so the false matches will lead to serious misalignment. TFT estimates a plane for every triangle instead of a homography for every cell, so TFT depends on the number of triangular facets in efficiency and runs faster than APAP generally. The warping-residual-based image stitching method [7] first estimates multiple homography matrices, and calculates warping residuals of each matched feature point using the multiple homography matrices. The homography of each region is estimated using moving DLT with the difference of the warping residuals as weight, which means the method can handle larger parallax than APAP, but is less robust to the incorrectly estimated homography and runs slower than APAP. The NIS (natural image stitching) [12] method estimates a pixel-to-pixel transformation based on feature matches and the depth map to achieve accurate local alignment. In [13], by increasing feature correspondences and optimizing hybrid terms, sufficient correct feature correspondences are obtained in the low-texture areas to eliminate misalignment. The two methods require additional runtime to enhance robustness, but also are susceptible to the uneven distribution and false matches of feature points.

For perspective deformation, SPHP (shape preserving half projective) [14,15] spatially combines perspective transformation and similarity transformation to reduce deformation. Perspective transformation can better align pixels in overlapping areas, and similarity transformation preserves the viewpoint of the original image in non-overlapping areas. AANAP (adaptive as-natural-as-possible) [16] derives the appropriate similarity transform directly based on matched feature points, and uses weights to gradually transit from perspective transform to similarity transform. The transitions from the homography of the overlapping area to the similarity matrix of the non-overlapping area adopted by SPHP and AANAP are artificial and unnatural, and can generate some “strange” homography matrices, causing significant distortion in the overlapping area. Both SPHP and AANAP require the estimation of homography or similarity matrices for each cell, and thus have the same efficiency issue as APAP. GSP (global similarity prior) [17,18] adds a global similarity prior to constrain the warping of each image so that it resembles a similarity transformation as a whole and avoids large perspective distortion. In SPW (single-projective warp) [19], the quasi-homography warp [20] is adopted to mitigate projective distortion and preserve the single perspective. SPSO (Structure Preservation and Seam Optimization) [4] uses a hybrid warping model based on multi-homography and mesh-based warp to obtain precise alignment of areas at different depths while preserving local and global image structures. GES-GSP (geometric structure preserving-global similarity prior) [21] employs deep learning-based edge detection to extract various types of large-scale edges, and further introduces large-scale geometric structure preservation to GSP to preserve the curves in images and protect them from distortion. GSP, SPW, SPSO and GES-GSP are based on content preserving warping and require constructing and solving a linear equation with m variables and n equations to acquire the corresponding coordinates after mesh warping, in which m is the number of cell vertices multiplied by 2, n is the number of alignment constraints, structural preservation constraints, and other constraints. Both m and n are generally larger, therefore more runtime is required.

Based on the above analysis, generating a natural mosaic quickly and robustly remains a challenging task.

## 3. Methodology

**Figure 1:**
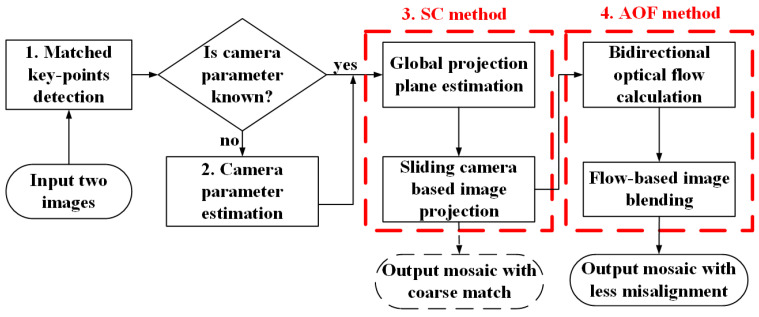
Flow chart of SC-AOF method. After the detection and matching of feature points, the camera parameters are obtained in advance or estimated. Then the two warped images are calculated using SC method, and the mosaic that is coarsely aligned can be obtained. Finally, the AOF method is used to further align the two warped images to generate a blended mosaic with higher alignment accuracy.

The flow chart of the SC-AOF algorithm is illustrated in Figure 1. The details on each of its steps are described below.

Step 1: Feature point detection and matching. SIFT (scale-invariant feature transform) and SURF (speed-up robust feature) methods are generally used to detect and describe key points from two input images. Using the KNN (k-nearest neighbor) method, a group of matched points is extracted from the key points and used for camera parameter estimation in step 2 and global projection plane calculation in step 3. 

Step 2: Camera parameter estimation. The intrinsic and extrinsic camera parameters are the basis of the SC method, and can be obtained in advance or estimated. When camera parameters are known, we can skip step 1 and directly start from step 3. When camera parameters are unknown, they can be estimated by minimizing the epipolar and planar errors, as described in Section 3.3.

Step 3: Sliding camera-based image projection. In this step, we estimate the global projection plane first, then adjust the camera projection matrix and generate a virtual camera in the overlapping area by interpolation, and obtain the warped images by global planar projection, as detailed in Section 3.1. Misalignment can be found in the two warped images obtained in the current step. Therefore, we need to use the AOF method in step 4 to further improve the alignment accuracy. 

Step 4: Flow-based image blending. In this step, we first calculate the bidirectional asymmetric optical flow between the two warped images, then further align and blend the two warped images to generate a mosaic using the optical flow (see Section 3.2 for more details).

### 3.1. SC: Viewpoint Preservation Based on Sliding Camera

The sliding camera (SC) method is proposed for the first time to solve perspective deformation, and is the first step in the SC-AOF method. For this reason, this section will first introduce the stitching process of this method, and then detail how to calculate the global projection plane and the sliding projection matrix required by this method.

#### 3.1.1. SC Stitching Process

In order to ensure that the mosaic can maintain the perspective of the two input images, the SC method is used. That is, in the non-overlapping area, the viewpoints of the two input images are preserved. In the overlapping area, the viewpoint of the camera is gradually transformed from $I_{1}$ to $I_{2}$.

**Figure 2:**
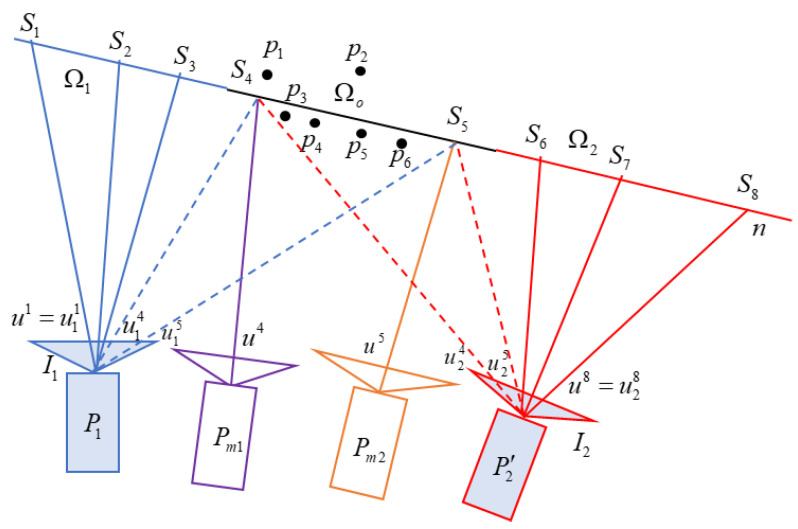
Image stitching based on sliding cameras. n is the projection surface, which is fitted by scene points $p_{1},p_{2},\ldots ,p_{6}$. Stitched image I can be generated by projection of sampling points $S_{1},S_{2},\ldots ,S_{8}$. The points $S_{1},S_{2},S_{3}$ in the area $\Omega_{1}$ are generated by back-projection of pixels in $I_{1}$. Similarly, the points $S_{6},S_{7},S_{8}$ in the area $\Omega_{2}$ are generated by back-projection of pixels in $I_{2}$. The points $S_{4},S_{5}$ in the area $\Omega_{o}$ are generated by back-projection of pixels in virtual cameras. The pixel values of $S_{4},S_{5}$ correspond to the fused pixel values of projection in $I_{1}$ and $I_{2}$. $P_{1}\;and\;P_{2}$ are the camera projection matrices of images $I_{1}\;and\;I_{2}$. To unify the pixel coordinates of $I_{1}$ and $I_{2}$, $P_{2}$ is adjusted to $P_{2}^{\prime }$ using the method in Section 3.1.3.

As shown in Figure 2, the image $I_{1}$ and $I_{2}$ are back-projected onto the projection surface n, so that the corresponding non-overlapping areas $\Omega_{1}$, $\Omega_{2}$ and overlapping area $\Omega_{o}$ are obtained. Assume that the pixels in the mosaic I are $u^{1},u^{2},\ldots ,u^{8}$, which correspond to the sampling points $S_{1},S_{2},\ldots ,S_{8}$ on the projection surface n. When the sampling points are within the projection area $\Omega_{1}$ of image $\;I_{1}$, the mosaic is generated from the viewpoint of $I_{1}$.$S_{1},S_{2},S_{3}$ are the intersection points of the backprojection lines of $u^{1},u^{2},u^{3}$ in $I_{1}$ and the projection surface n. Therefore, $u^{i}=P_{1}S_{i}\left(i=1,\;2,\;3\right)$, where $P_{1}$ is the projection matrix of $I_{1}$. When the sampling points are within the projection area $\Omega_{2}$ of image $I_{2}$, the mosaic is generated from the camera viewpoint of $I_{2}$. Similarly, we obtain $S_{i}$ and $u^{i}=P_{2}S_{i}$, where i = 6, 7, 8. In the overlapping area $\Omega_{o}$ of $I_{1}$ and $I_{2}$, the SC method is used to generate a virtual camera, whose viewpoint gradually transitions from the viewpoint of $I_{1}$ to that of $I_{2}$. $S_{4}$ and $S_{5}$ are the intersection points of the back-projection lines of $u^{4}$, $u^{5}$ in the visual camera and projection plane n, respectively. The virtual camera’s image is generated from images $I_{1}$ and $I_{2}$ using perspective transformation. For example, pixel $u^{4}$ of the virtual camera corresponds to pixel $u_{1}^{4}$ in $I_{1}$ and pixel $u_{2}^{4}$ in $I_{2}$, and are generated by blending the latter two pixels.

**Global projection surface calculation.** In order to match the corresponding pixels $u_{1}^{4}$ of $I_{1}$ and $u_{2}^{4}$ of $I_{2}$, the projection surface n needs to be as close as possible to the real scene point; we can use the moving plane method [7,8,9] or the triangulation method [6] to obtain a more accurate scene surface. Since the SC-AOF method will use the optical flow to further align the images, for the stitching speed and stability, only the global plane is calculated as the projection surface. Section 3.1.2 will calculate the optimal global projection surface using the matched points.

**Sliding camera generation.** Generally, since the pixel coordinates of $I_{1}$ and $I_{2}$ are not uniform, in the mosaic I, when $I(\tilde{u})=I_{1}(P_{1}S)$ in the non-overlapping area of $I_{1}$, $I(\tilde{u})=I_{2}(P_{2}S)$ is false in the non-overlapping area of $I_{2}$, where S is the sampling point on the projection surface. It is necessary to adjust the projection matrix of $I_{2}$ to $P_{2}^{\prime }$, so that $I(\tilde{u})=I_{2}(P_{2}^{\prime }S)$. The red camera is shown in Figure 2. Section 3.1.3 will deduce the adjustment method of the camera projection matrix, and interpolate in the overlapping area to generate a sliding camera, and obtain the warped images of $I_{1}$ and $I_{2}$.

#### 3.1.2. Global Projection Surface Calculation

The projection matrices of cameras $C_{1}$ and $C_{2}$ corresponding to images $I_{1}$ and $I_{2}$ are:

(1) $$P_{1}=K_{1}[I_{3\times 3}|0]\;P_{2}=K_{2}R[I_{3\times 3}|-t]$$

where $K_{1}$ and $K_{2}$ are the intrinsic parameter matrices of $C_{1}$ and $C_{2}$ respectively; R is the inter-camera rotation matrix; and t is location of the optical center of $C_{2}$ in the coordinate system $C_{1}$.

The relationship between the projection $u_{1}$ in $I_{1}$ and the projection $u_{2}$ in $I_{2}$ of a 3D point p on plane n is:

(2) $${\tilde{u}}_{2}∼K_{2}R(I_{3\times 3}+tn^{T})K_{1}^{-1}{\tilde{u}}_{1}=H{\tilde{u}}_{1}$$

where ${\tilde{u}}_{1}$ and ${\tilde{u}}_{2}$ are the homogeneous coordinates of $u_{1}$ and $u_{2}$, respectively. The intersection point p satisfies $n^{T}p+1=0$. ∼ means that ${\tilde{u}}_{2}$ is parallel to $H{\tilde{u}}_{1}$.

If camera parameters $K_{1},K_{2}$, R and t are known, then we can deduce the following Equation (3) from Equation (2)

(3) $$n^{T}{\tilde{y}}_{1}=-\frac{\left(R^{T}{\tilde{y}}_{2}\times t{)}^{T}(R^{T}{\tilde{y}}_{2}\times {\tilde{y}}_{1}\right)}{\left(R^{T}{\tilde{y}}_{2}\times t{)}^{T}(R^{T}{\tilde{y}}_{2}\times t\right)}=b$$

where ${\tilde{y}}_{1}=K_{1}^{-1}{\tilde{u}}_{1}$ and ${\tilde{y}}_{2}=K_{2}^{-1}{\tilde{u}}_{2}$ are the normalized coordinates of ${\tilde{u}}_{1}$ and ${\tilde{u}}_{2}$, respectively.

We use Equation (3) of all matched points to construct an overdetermined equation and obtain the fitted global plane n by solving this equation. Since the optical-flow-based stitching method will be used to further align the images, the RANSAC method is not used here to calculate the plane with the most inliers. Instead, the global plane that fits all feature points as closely as possible is selected, misalignment caused by global plane projection will be better solved during optical flow blending.

#### 3.1.3. Projection Matrix Adjustment and Sliding Camera Generation

To preserve the viewpoint in the non-blending area of $I_{2}$, it is only required to satisfy $I\left(\tilde{u}\right)=I_{2}\left(N\tilde{u}\right)=I_{2}\left({\tilde{u}}_{2}\right)$, where $\tilde{u}$ is the homogeneous coordinate of a pixel in the mosaic, ${\tilde{u}}_{2}\;is\;the\;homogeneous\;coordinate\;of\;a\;pixel\;in\;I_{2},\;N$ is a similarity transformation between $I_{2}$ and I, and can be obtained by fitting the matched feature points:

(4) $$N_{*}=\underset{S}{min}\sum_{j=1}^{n}||N{\tilde{u}}_{1}^{j}-{\tilde{u}}_{2}^{j}{\left|\right|}^{2}$$

where ${\tilde{u}}_{1}^{j}$ and ${\tilde{u}}_{2}^{j}$ are the homogeneous coordinates of pixels in $I_{1}$ and $I_{2}$ respectively.

Therefore, in the non-overlapping area of $I_{2}$, $\tilde{u}=N_{*}^{-1}{\tilde{u}}_{2}=N_{*}^{-1}K_{2}R_{2}(S-t)$, where S is the corresponding 3D point of ${\tilde{u}}_{2}$ on plane n. So we get the projection matrix $P_{2}^{\prime }$=$N_{*}^{-1}K_{2}R_{2}$.

By RQ decomposition, the internal parameter matrix $K_{2}^{\prime }$ and rotation $R_{2}^{\prime }$ are extracted from $P_{2}^{\prime }$:

(5) $$N_{*}^{-1}K_{2}R_{2}=K_{2}\left(K_{2}^{-1}N_{*}^{-1}K_{2}\right)R_{2}=K_{2}\left(K_{*}R_{*}\right)R_{2}=K_{2}^{\prime }R_{2}^{\prime }$$

where $K_{2}^{\prime }$ and $R_{2}^{\prime }$ are upper triangular matrix and rotation matrix respectively; and the third line of both matrices is $\left(\begin{array}{ccc} 0 & 0 & 1 \end{array}\right)$.

Compared with $P_{2}$, $P_{2}^{\prime }$ has a different intrinsic parameter matrix, and its rotation matrix only differs by one rotation around Z axis, and its optical center t is not changed.

(6) $$t_{m}=(1-m)0_{3\times 1}+mt$$

(7) $$K_{m}=(1-m)K_{1}+mK_{2}^{\prime }$$

(8) $$q_{m}=\frac{\sin((1-m)\theta )}{\sin(\theta )}q_{1}+\frac{\sin(m\theta )}{\sin(\theta )}q_{2}$$

where $q_{1},q_{2},q_{m}$ represent the quaternions corresponding to $I_{3\times 3}$, $R_{2}^{\prime }$ and $R_{m}$, θ is the angle between $q_{1}$ and $q_{2}$, and m is the weighting coefficient.

**Figure 3:**
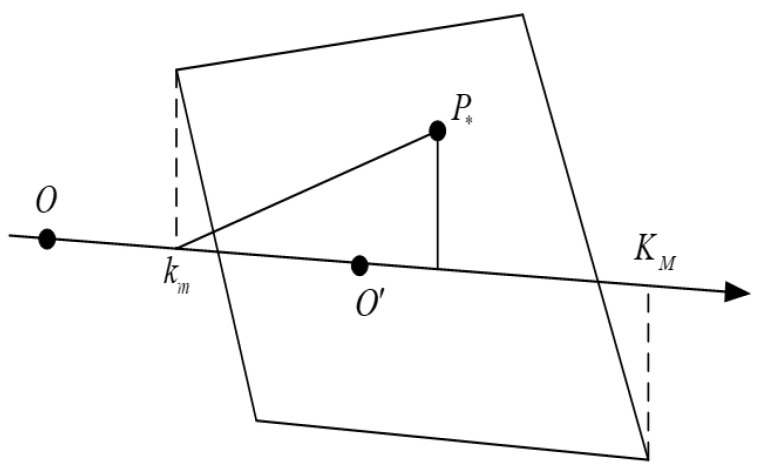
The diagram of gradient weight. The quadrilateral is the boundary of the overlapping area of $I_{1}$ and mapped image of $I_{2}$ using $H^{-1}$, where O is the center of $I_{1}$ and $O^{\prime }$ is the warped point of the center point of $I_{2}$ using $H^{-1}$. $k_{m}$ and $K_{M}$ are the projection points closest to O and $O^{\prime }$ on the line $OO^{\prime }$ of the quadrilateral vertices, respectively. $P_{*}$ indicates the pixel coordinates within the overlapping area that need to calculate weighted parameter m.

As depicted by Figure 3, the weighting coefficient m can be calculated by the method in AANAP [16]:

(9) $$m=\left\langle \vec{k_{m}P_{*}},\vec{k_{m}K_{M}}\right\rangle /|\vec{k_{m}K_{M}}{|}^{2}$$

In the overlapping area, if u corresponds to sliding camera $\left(K_{m},R_{m},t_{m}\right)$, then the relation between u and $u_{i}$ in $I_{i}(i=1,2)$ can be expressed as:

(10) $$\tilde{u}=K_{m}R_{m}(I+t_{m}n^{T}/d)K_{1}^{-1}{\tilde{u}}_{1}=H_{m}^{1}{\tilde{u}}_{1}$$

(11) $$\tilde{u}=H_{m}^{1}H^{-1}{\tilde{u}}_{2}=H_{m}^{2}{\tilde{u}}_{2}$$

Equations (10) and (11) are also applicable to the non-overlapping area. Projecting $I_{1}$ and $I_{2}$ through $H_{m}^{1}$ and $H_{m}^{2}$ onto the mosaic, respectively, to get warped images $I_{1}^{\prime }$ and $I_{2}^{\prime }$. Obviously:

(12) $$I_{i}^{\prime }(\tilde{u})=I_{i}((H_{m}^{i}{)}^{-1}\tilde{u})(i=1,2)$$

**Figure 4:**
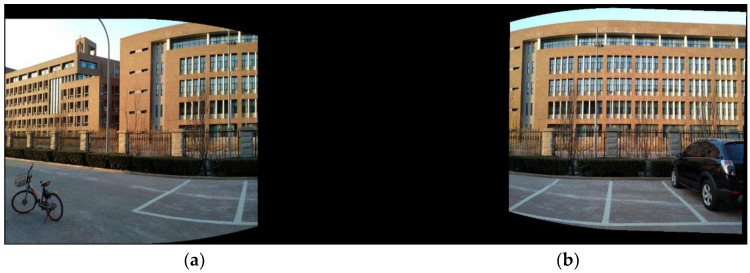

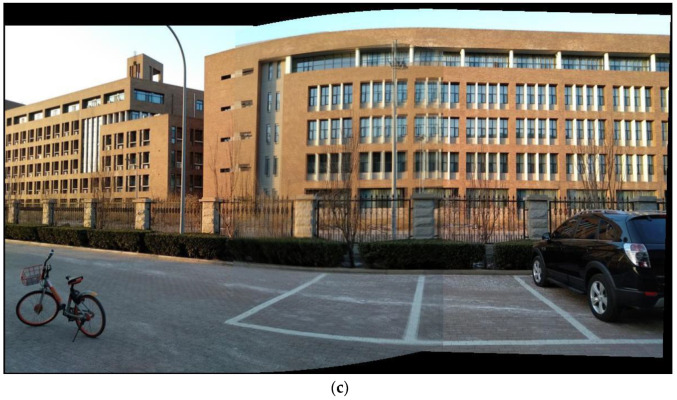
Image stitching based on sliding cameras and global projection plane. (**a**,**b**) show the warped images $I_{1}^{\prime }$ and $I_{2}^{\prime }$ of the input images of a school; (**c**) shows the average blending images of $I_{1}^{\prime }$ and $I_{2}^{\prime }$. That is, in the overlapping area, the blended value is $(I_{1}^{\prime }+I_{2}^{\prime })/2$.

Figure 4 shows the experiment result on two school images used in [10]. Due to the parallax between $I_{1}$ and $I_{2}$, blending $I_{1}^{\prime }$ and $I_{2}^{\prime }$ will cause ghosting. Therefore, the next section will use an optical-flow-based blending method (AOF) to further align the images.

### 3.2. AOF: Image Alignment Based on Asymmetric Optical Flow

The mosaic generated by the SC method will inevitably have misalignment in most cases. So, the optical-flow-based method is further employed to achieve more accurate alignment. This section firstly introduces the image alignment process based on asymmetric optical flow (AOF), and then details the calculation method of AOF.

#### 3.2.1. Image Blending Process of AOF

**Figure 5:**
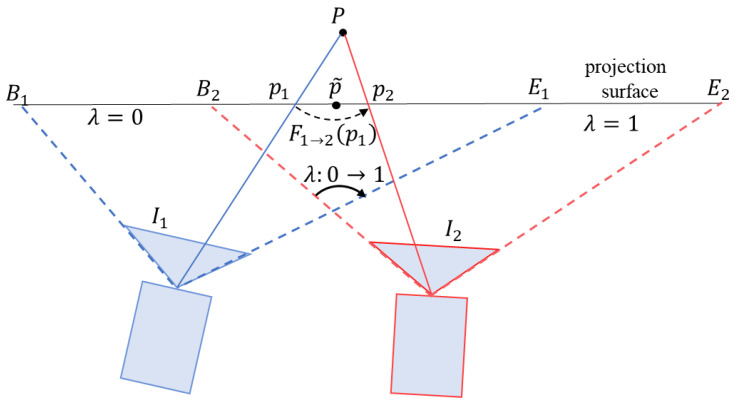
Image blending based on optical flow. $B_{1}E_{2}$ is the projection surface of the mosaic. In the overlapping areas (denoted by $B_{2}E_{1}$) of $I_{1}$ and $I_{2}$, we need to blend $I_{1}^{\prime }$ and $I_{2}^{\prime }$. The 3D point P is outside the projection surface. When P is projected onto the projection surface, ghosting points $p_{1}$ and $p_{2}$ appear. Through the weighted blending of asymmetric optical flow, $p_{1}$ and $p_{2}$ are merged into point $\tilde{p}$, which solves the ghosting problem of stitching.

$I_{1}$ and $I_{2}$ are projected onto the custom projection surface to obtain warped images $I_{1}^{\prime }$ and $I_{2}^{\prime }$, which are then blended to generate the mosaic I. As the 3D points of the scene are not always on the projection plane, ghosting artifacts can be seen in the mosaic, as shown in Figure 4 in the previous section. Direct multi-band image blending will lead to artifacts and blurring. As shown in Figure 5, point P is projected to two points $p_{1}$ and $p_{2}$ in the mosaic, resulting in duplication of content. To solve the ghosting problem in the mosaic, the optical-flow-based blending method in [22] is adopted.

Suppose $F_{2\to 1}\left(p_{2}\right)$ represents the optical flow value of $p_{2}$ in $I_{2}^{\prime }$ and $F_{1\to 2}\left(p_{1}\right)$ represents the optical flow value of $p_{1}$ in $I_{1}^{\prime }$. If the blending weight of pixel $\tilde{p}$ in the overlapping area is λ (from the non-overlapping area of $I_{1}^{\prime }$ to the non-overlapping area of $I_{2}^{\prime }$), λ gradually transitions from 0 to 1, as shown in Figure 5, then after blending, the pixel value of image I at $\tilde{p}$ is:

(13) $$I\left(\tilde{p}\right)=\left(1-\lambda \right)I_{1}^{\prime }\left({\tilde{p}}_{1}\right)+\lambda I_{2}^{\prime }({\tilde{p}}_{2})$$

where ${\tilde{p}}_{1}=\tilde{p}+\lambda F_{2\to 1}\left(\tilde{p}\right)$ represents the corresponding value of $\tilde{p}$ in $I_{1}^{\prime }$, and ${\tilde{p}}_{2}=\tilde{p}+\left(1-\lambda \right)F_{1\to 2}\left(\tilde{p}\right)$ represents the corresponding value of $\tilde{p}$ in $I_{2}^{\prime }$. That is, for any pixel $\tilde{p}$ in the overlapping area of the mosaic, its final pixel value can be obtained by a weighted combination of its corresponding values in the two warped images using optical flow.

To achieve get a better blending effect, following the method presented by Meng and Liu [23], a softmax function is used to facilitate the mosaic transition quickly from $I_{1}^{\prime }$ to $I_{2}^{\prime }$, narrowing the blending area. Furthermore, if the optical flow value of a warped image is larger, the salience is higher, and the blending weight of the warped image should be increased accordingly. Therefore, the following blending weight β can be employed:

(14) $$\beta =\frac{\exp(\alpha_{s}\lambda (1+\alpha_{m}M_{2}))}{\exp(\alpha_{s}(1-\lambda )(1+\alpha_{m}M_{1}))+\exp(\alpha_{s}\lambda (1+\alpha_{m}M_{2}))}$$

where $M_{1}=||F_{2\to 1}(\tilde{p})||$ and $M_{2}=||F_{1\to 2}(\tilde{p})||$ represents the optical flow magnitude; $\alpha_{s}$ is the shape coefficient of the softmax function; and $\alpha_{m}$ denotes the enhancement coefficient of the optical flow. The larger $\alpha_{s}$ and $\alpha_{m}$ are, the closer β is to 0 or 1, and the smaller the image transition area becomes.

Also, similar to multi-band blending, a wider blending area is used in smooth and color-consistent areas, and a narrower blending area is used in color-inconsistent areas. And the pixel consistency is measured using $D_{c}$:

(15) $$D_{c}=||I_{1}({\tilde{p}}_{1})-I_{2}({\tilde{p}}_{2})||$$

The final blending parameter α is obtained:

(16) $$\lambda_{d}=tanh\;(c_{d}D_{c})$$

(17) $$\alpha =(1-\lambda_{d})\lambda +\lambda_{d}\beta$$

β corresponds to a fast transition from $I_{1}^{\prime }$ to $I_{2}^{\prime }$, λ corresponds to a linear transition from $I_{1}^{\prime }$ to $I_{2}^{\prime }$. When the color differs slightly, the transition from $I_{1}^{\prime }$ to $I_{2}^{\prime }$ is linear, and when the color difference is large, we tend to have a fast transition from $I_{1}^{\prime }$ to $I_{2}^{\prime }$.

Then the pixel value of the mosaic is:

(18) $$I(\tilde{p})=(1-\alpha )I_{1}({\tilde{p}}_{1})+\alpha I_{2}({\tilde{p}}_{2})$$

**Figure 6:**
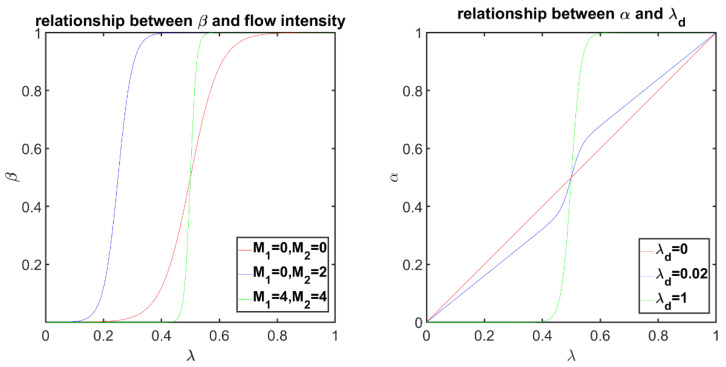
Blending parameter curves. The figure on the left shows the β curves at different optical flow intensities. The right figure shows the α curve at different $\lambda_{d}$ values.

The curve in the left panel of Figure 6 shows the curve of β with respect to λ under different optical flow intensities. β can be used to achieve quick transition of the mosaic from $I_{1}^{\prime }$ to $I_{2}^{\prime }$, narrowing the transition area. In the case of a large optical flow, the blending weight of the corresponding image can be increased to reduce the transition area. The curve in the right panel of Figure 6 shows the influence of $\lambda_{d}$ on the curve of α as a function of λ. When $\lambda_{d}$ is small, a wider fusion area tends to be used; otherwise, a narrower fusion area is used, which is similar to the blending of different frequency bands in a multi-band blending method.

#### 3.2.2. Calculation of Asymmetric Optical Flow

The general pipeline of the optical flow calculation is to construct an image pyramid, calculate the optical flow of each layer from coarse to fine, and use the estimated current-layer optical flow divided by the scaling factor as the initial optical flow of the finer layer until the optical flow of the finest layer is obtained [23,24,25,26]. Different methods are proposed to achieve better solutions that satisfy brightness constancy assumptions, solve large displacements and appearance variation [27,28], and address edge blur and improve temporal consistency [29,30,31]. Recently, some deep learning methods have been proposed. For example, RAFT (recurrent all-pairs field transforms for optical flow) [32] extracts per-pixel features, builds multi-scale 4D correlation volumes for all pairs of pixels, and iteratively updates a flow field through a recurrent unit. FlowFormer (optical flow Transformer) [33] is based on a transformer neural network architecture with a novel encoder which effectively aggregates cost information of correlation volume into compact latent cost tokens, and a recurrent cost decoder which recurrently decodes cost features to iteratively refine the estimated optical flows.

In order to improve the optical flow calculation speed, we use the method based on optical flow propagation and gradient descent adopted in Facebook surround360 [34] to calculate the optical flow. When calculating the optical flow of each layer, first calculate the optical flow of each pixel from top to bottom and from left to right. From the optical flow values of the current-layer left and top pixels and upper-layer same-position pixel, the value with minimum error represented by Equation (19) is selected as the initial value of the current pixel. Then a gradient descent method is performed to update the optical flow value of the current pixel, and is then spread to the right and bottom pixels, as a candidate for the initial optical flow of the right and bottom pixels. After completing the forward optical flow propagation from top to bottom and from left to right, perform a reverse optical flow propagation and gradient descent from bottom to top and from right to left to obtain the final optical flow value.

When calculating the optical flow value F(u) of pixel u, the error function E(F(u)) used is:

(19) $$E(F(u))=E_{I}+\alpha_{S}E_{S}+\alpha_{T}E_{T}$$

(20) $$E_{I}(F(u))=||\nabla I_{1}(u)-\nabla I_{2}(u+F(u))||$$

(21) $$E_{S}(F(u))=||F(u)-G(u;\sigma )*F(u)||$$

(22) $$E_{T}(F(u))=||F(u)*D(1/W,1/H)||$$

where $E_{I}$ denotes the optical flow alignment error of the edge image (which is Gaussian filtered to improve the robustness); $E_{S}$ denotes the consistency error of the optical flow; G(u;σ)∗F(u) denotes the Gaussian-filtered optical flow of pixel u; $E_{T}$ denotes the magnitude error after normalization of optical flow, with excessively large optical flow being penalized; W and H are the width and height of the current-layer image, respectively, D(1/W,1/H) denotes the diagonal matrix with diagonal elements 1/W and 1/H.

### 3.3. Estimation of Image Intrinsic and Extrinsic Parameters

The SC-AOF method requires known camera parameters of images $I_{1}$ and $I_{2}$. When only the intrinsic parameters $K_{1}$ and $K_{2}$ of an image are known, the essential matrix ${\left[t\right]}_{\times }R$ between two images can be obtained by feature point matching, and the rotation matrix R and translation vector t between images can be obtained by decomposing the essential matrix. When both intrinsic and extrinsic parameters are unknown, the intrinsic parameters can be estimated by calibration [35,36] firstly, and then the extrinsic parameters of the image can be estimated accordingly. In these cases, both intrinsic and extrinsic parameters of image $I_{1}$ and $I_{2}$ can be estimated robustly.

When none of the above methods is feasible, it is necessary to calculate the fundamental matrix from the matched feature points and restore the camera internal and external parameters.

When the camera has zero skew, the known principal point and aspect ratio, then each intrinsic parameter matrix has only one degree of freedom (focal length of the camera). The total degree of freedom of the camera parameters is 7 (where t has 2 degrees of freedom due to the inability to recover scale, R has 3 degrees of freedom, and each camera has 1 degree of freedom), which is equal to the fundamental matrix F’s degree of freedom. The internal and external parameters of the image can be recovered using a self-calibration method [37]. But even if these constraints are met, the camera parameters by solved [37] suffer from large errors when the scene is approximately planar and the matching error is large. Therefore, we use the method of optimizing the objective function in [6] to solve the internal and external parameters of the camera. 

**Figure 7:**
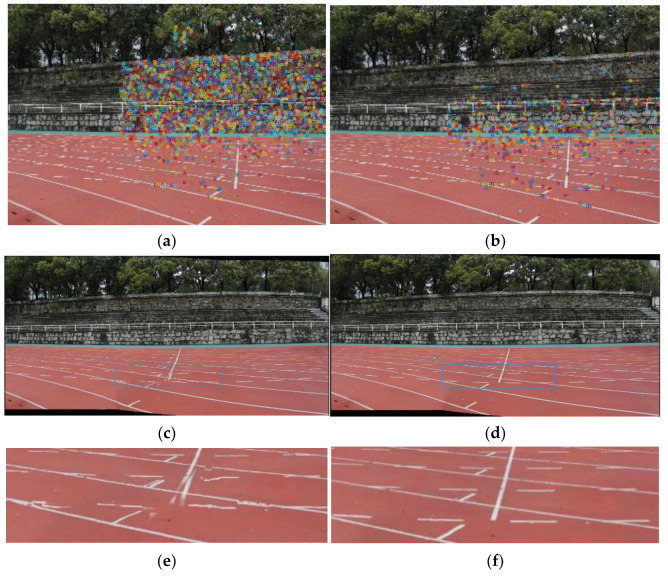
The impact of feature point distribution on stitching results. The feature points are marked by small color circles, and the blue boxes indicate the regions where the enlarged images are located in the mosaics. The feature points in (**a**) are concentrated in the grandstand. The corresponding mosaic (**c**) is misaligned in the playground area. The feature points in (**b**) are evenly distributed within a 2×2 grid. Although the total number of feature points is smaller, the mosaic (**d**) has better quality. (**e**,**f**) show the detail of mosaics.

To obtain an accurate fundamental matrix, firstly, the feature points need to be distributed more evenly in the image. As shown in Figure 7, a uniform and sparse distribution of feature points can both reduce the computation time and obtain more robust intrinsic and extrinsic camera parameters and global projection planes, which will lead to improved stitching results.

Secondly, it is necessary to filter the matched feature points to exclude the influence of outliers. Use the similarity transformation to normalize the matched feature points. After normalization, the mean value of the feature points is 0, and the average distance to the origin is $\sqrt{2}$. 

Thirdly, multiple homographies are estimated to exclude outlier points. Let $F_{cond}$ and n denote all matched feature points and the total number of matched feature points. In $F_{cond}$, the RANSAC method with threshold η=0.01 is applied to compute homography $H^{1}$ and its inlier set $F_{inlier}^{1}$, and the matches of isolated feature points which have no neighboring points within a 50 pixel distance are removed from $F_{inlier}^{1}$. A new candidate set is generated by removing $F_{inlier}^{1}$ from $F_{cond}$. Repeat the above steps to calculate m homography matrices $H^{m}$ and corresponding inlier set $F_{inlier}^{m}$ until $||F_{inlier}^{m}||<$20 or $|F_{inlier}^{m}||<0.05n$. The final inlier set is $F_{inlier}=\cup_{i=1}^{m}F_{inlier}^{i}$. If m=1, then there is only one valid plane. In this case, apply the RANSAC method with threshold η=0.1 to recalculate homography $H^{1}$ and the corresponding inlier set $F_{inlier}^{1}$.

After excluding the outliers, for any matched points $\left\{x_{1},x_{2}\right\}$ in the inlier set $F_{inlier}$, the cost function is:

(23) $$E(x_{1},x_{2})=\left(1-\lambda \right)h(r_{e},\sigma_{e})+\lambda h(r_{p},\sigma_{p})$$

where λ balanced epipolar constraint and the infinite homography constraint, and generally take λ=0.01. h is a robust kernel function, which can mitigate the effect of mis-matched points on the optimization of camera internal and external parameters. $r_{e}$ and $r_{p}$ denote the projection errors of the epipolar constraint and of the infinite homography constraint, respectively:

(24) $$r_{e}=\frac{1}{\rho }x_{2}^{T}K_{2}^{-T}[t{]}_{\times }RK_{1}^{-1}x_{1}=\frac{1}{\rho }x_{2}^{T}Fx_{1}$$

(25) $$r_{p}=x_{2}-\frac{1}{\omega }K_{2}RK_{1}^{-1}x_{1}$$

where ρ denotes the length of the vector composed of the first two components of $Fx_{1}$. That is, assuming $Fx_{1}=(a,b,c{)}^{T}$, then $\rho =\sqrt{a^{2}+b^{2}}$. w represents the third component value of the vector $K_{2}RK_{1}^{-1}x_{1}$.

## 4. Experiment

**Figure 8:**
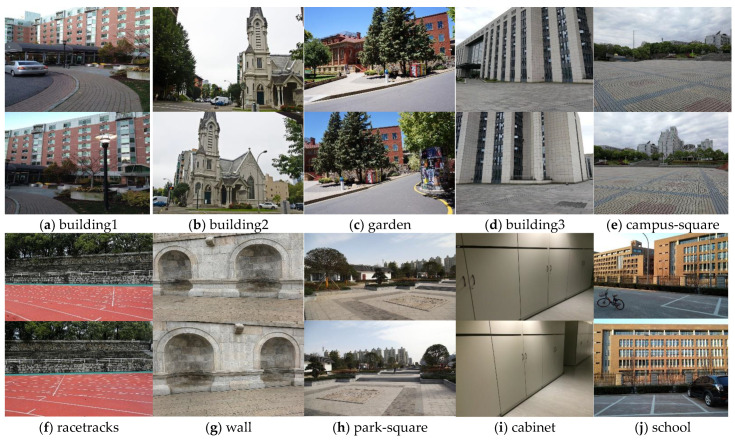
The image dataset for comparative experiments. The image pairs are initially used by stitching methods such as APAP, AANAP, and REW.

To verify the effectiveness of the SC-AOF method, the mosaics generated by our method and the existing APAP [4], AANAP [16], SPHP [14], TFT [7], REW [10] and SPW [18] methods are compared on some typical datasets used by others to verify the feasibility and advantages of the SC-AOF method in solving deformation and improving alignment. Next, the SC-AOF method is used together with other methods to demonstrate its compatibility. The image pairs used in the comparison experiment are shown in Figure 8.

### 4.1. Effectiveness Analysis of SC-AOF Method

In this section, various methods of image stitching are compared and analyzed based on three indicators: perspective deformation, local alignment and running speed. The experimental setup is as follows.

- The first two experiments compare typical methods for solving perspective deformation and local alignment, respectively, and all the methods in the first two experiments are included in the third experiment to show the superiority of the SC-AOF method in all aspects.
- Since the averaging methods generally underperform compared to linear blending ones, all methods to be compared adopt linear blending to achieve the best performance.
- All methods other than ours use the parameters recommended by their proposers. Our SC-AOF method has the following parameter settings in optical-flow-based image blending: $\alpha_{s}=$ 10, $\alpha_{m}=$ 100, and $c_{d}=$ 10.

#### 4.1.1. Perspective Deformation Reduction

**Figure 9:**
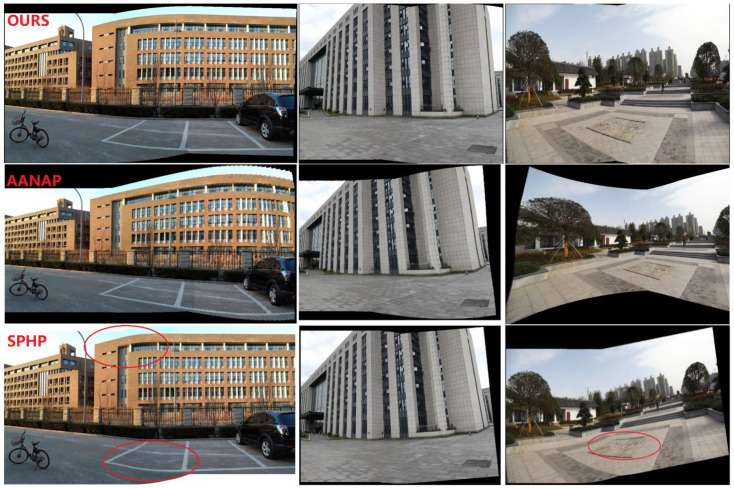

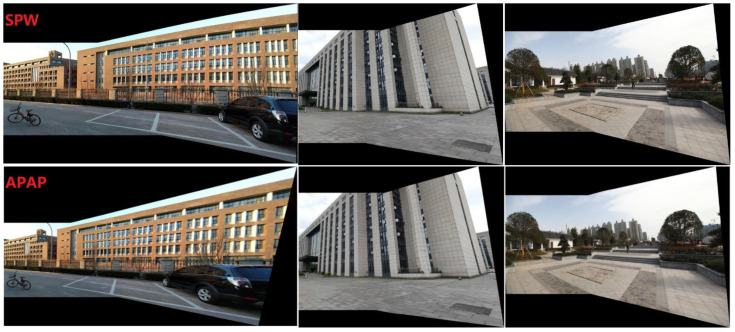
Comparison of perspective deformation processing. From the first row to the last row, the mosaics generated by our method, AANAP, SPHP, SPW and APAP on the datasets are presented, respectively. The red elliptical boxes indicate the unnatural transitions in the mosaics.

Figure 9 shows the results of the SC-AOF method versus the SPHP, APAP, AANAP and SPW methods for perspective deformation reduction in image stitching. School, building and park square datasets were used in this experiment. We can see from Figure 9 that, compared with the other methods, our SC-AOF method changes the viewpoint of the stitched image in a more natural manner and effectively eliminates perspective deformation. As explained below, all other methods underperform compared to our SC-AOF method.

The image stitched using the APAP method has its edges stretched to a large extent. This is because it does not process perspective deformation. This method only serves as a reference to verify the effectiveness of perspective-deformation-reducing algorithms.

The AANAP algorithm can achieve a smooth transition between the two viewpoints, but results in severely “curved edges”. And there is even more severe edge stretching for the park square dataset than that of the APAP method. This is because, when the AANAP method extrapolates from homographies, it linearizes the homography in addition to similarity transformation, causing affine deformation in the final transformation.

Compared with the APAP method, the SPW method makes no significant improvement in perspective deformation, except for the image in the first row. SPW preserves perspective consistency, so a multiple-viewpoint method excels in solving perspective deformation compared to single-viewpoint method.

The SPHP algorithm performs well overall. However, it causes severe distortions in some areas (red circles in Figure 8c) due to the rapid change of viewpoints. This is because the SPHP method estimates the similarity transformation and interpolated homographies from global homography. As a result, the similarity transformation cannot reflect the real scene information and the interpolated homographies may deviate from a reasonable image projection.

#### 4.1.2. Local Alignment

**Figure 10:**
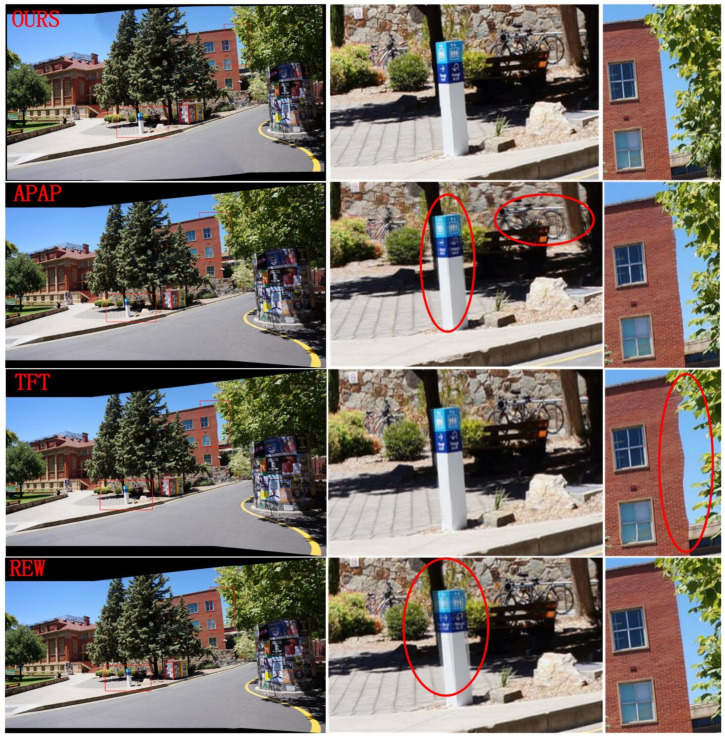
Qualitative comparison on the garden image pairs. From the first row to the last row, the mosaics and detail views generated by our method, APAP, TFT, REW are presented, respectively. The red boxes indicate the regions where the enlarged images are located in the mosaics. The red circles highlight errors and distortions.

**Figure 11:**
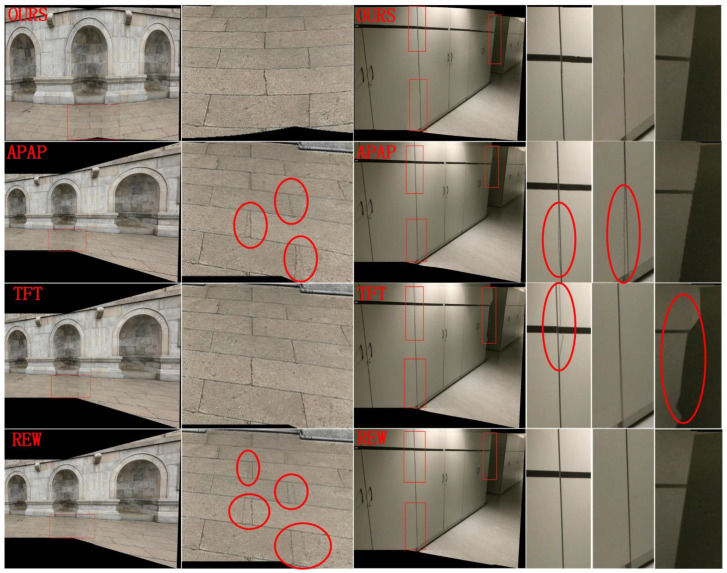
Comparison of image alignment on the wall and cabinet image pairs. From the first row to the last row, the mosaics and detail views generated by our method, APAP, TFT, REW are presented, respectively. The blue boxes indicate the region where the enlarged images are located. The red circles highlight errors and distortions.

Figure 10 and Figure 11 show the results of the SC-AOF method versus APAP, TFT and REW methods for local alignment in image stitching. It can be seen that SC-AOF performs well in all scenes, showing the effectiveness of our method in local alignment.

- The APAP method performs fairly well in most images, though with some alignment errors. This is because the moving DLT method smooths the mosaics to some extent.
- The TFT-generated stitched image is of excellent quality in planar areas. But when there is a sudden depth change in the scene, there are serious distortions. This is because large errors appear when calculating planes using three vertices of a triangle in the area with sudden depth changes.
- The REW method has large alignment errors in the planar area and aligns the images better than the APAP and TFT method in all other scenes. This is because the fewer feature points in the planar area might be filtered out as mismatched points by the REW method.

The SSIM (structural similarity) [38] is employed to objectively describe the alignment accuracy of different methods. SSIM measures the similarity between two images $J_{1}$ and $J_{2}$ to be blended in the overlapping area. For our two-step alignment method, $J_{1}$(u) = $I_{1}^{\prime }\left(u+\lambda F_{2\to 1}\left(u\right)\right)$, $J_{2}$(u) = $I_{2}^{\prime }\left(u+(1-\lambda )F_{1\to 2}\left(u\right)\right)$. The structural similarity is defined as:

(26) $$SSIM(J_{1},J_{2})=\frac{\left(2\mu_{1}\mu_{2}+C_{1})(2\sigma_{12}+C_{2}\right)}{{(\mu }_{1}^{2}+\mu_{2}^{2}+C_{1})(\sigma_{1}^{2}+\sigma_{2}^{2}+C_{2})}$$

where $\mu_{1}$ and $\sigma_{1}$ represent the mean and standard deviation of pixel values within the overlapping area O of $J_{1}$, respectively. $\mu_{2}$ and $\sigma_{2}$ are the corresponding mean and standard deviation of $J_{2}$, respectively. $\sigma_{12}$ is the covariance of pixel values in the overlapping area of $J_{1}$ and $J_{2}$. $C_{1}={\left(k_{1}L\right)}^{2}$ and $C_{2}={\left(k_{2}L\right)}^{2}$ are constants used to maintain stability, where $k_{1}=0.01$, $k_{1}=0.03$, and *L* is the dynamic range of pixel values (for 8-bit grayscale images, L=255). 

The scores of all methods on the datasets building1, building2, garden, building, school, park-square, wall, cabinet, campus-square and racetracks are listed in Table 1. The best SSIM value is highlighted in bold.

- APAP and AANAP have high scores on all image pairs, but the scores are lower than our method and REW, proving that APAP and AANAP blur mosaics to some extent.
- When SPHP is not combined with APAP, only the global homography is used to align the images, resulting in lower scores compared to other methods.
- TFT has higher scores on the datasets except for the building dataset. TFT can improve alignment accuracy but also bring instability.
- SPW combines quasi-homography and content-preserving warping to align images, which add other constraints while also reducing the accuracy of alignment, resulting in lower scores compared to REW and our method.
- Both REW and our method use a global homography matrix to coarsely align the images. Afterwards, in REW and our method, a deformation field and optical flow are applied to further align the images, respectively. Therefore, both methods have higher scores and robustness than other methods.

#### 4.1.3. Stitching Speed Comparison

**Figure 12:**
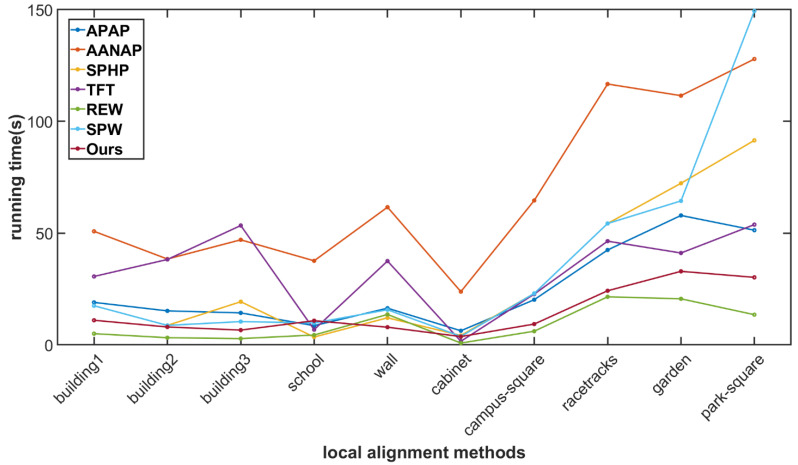
Comparison on elapsed time. Our method is second only to REW in speed and is superior to other methods.

The running speed is a direct reflection of the efficiency of each stitching method. Figure 12 shows the speed of the SC-AOF method versus the APAP, AANAP, SPHP, TFT, REW and SPW methods. The same image pairs as in the SSIM comparison are used in this experiment. It can be seen that the REW algorithm has the fastest stitching speed. The reason is that it only needs to calculate TPS parameters based on feature point matching and then compute the transformations of grid points quickly. Our SC-AOF method ranks second in terms of stitching speed, and the AANAP algorithm requires the longest running time. Both the APAP and AANAP methods calculate the local homographies based on moving DLT, and the AANAP method also needs to calculate the Taylor expansion of anchor points. 

#### 4.1.4. Overall Scoring for All the Methods

In order to comprehensively and quantitatively evaluate our method and other methods in improving local alignment and reducing perspective deformation, we define a scoring method that assigns an integer score ranging from 0 to 10 to estimate the effectiveness and efficiency of stitching each image pair using each method. The total score is obtained by adding up the scores from four aspects:

1. The subjective scoring of perspective deformation reduction. The scores from 0 to 2, respectively, indicate severe deformation, slight relief of deformation, and less deformation.
2. The subjective scoring of local alignment. The score ranges from 0 to 2, where 0 indicates obvious ghosting in many regions, 1 indicates few or mild mismatches, and 2 indicates no apparent alignment errors.
3. The objective scoring of local alignment. The score ranges from 0 to 3. We define the mean and standard deviation of the SSIM values of different methods on the same image pair as μ and σ, the SSIM of current method is x, the score of the method is 0, 1, 2 and 3, respectively, when x satisfies x−μ<−σ, $x-\mu \in \left(-\sigma ,0\right)$, $x-\mu \in \left(0,-\sigma \right)$ and x−μ>σ.
4. The scoring of running time. Like the objective scoring for local alignment, we score 0 when the running time of the method is greater than the mean plus standard deviation. When the time is less than the mean plus standard deviation and greater than the mean, the score is 1. The score is 2 when the time is less than the mean and greater than the mean minus standard deviation. Otherwise, the score is 3.

The scoring results of these methods on the image pairs are shown in Table 2. The image pairs in Table 2 include those used in SSIM and runtime comparison, as well as the test image pairs in the Appendix B (specific comparison of the mosaics generated by different methods are shown in the Appendix B). Every scoring is displayed in the format as “the score of perspective deformation reduction + the subjective score of local alignment + the objective score of local alignment + the score of running time = the overall score”. The highest score is bolded and highlighted. Our SC-AOF method has the highest scores in all the image pairs except the worktable image pair. Given that our method and REW all scored highly and have the same scores on some image pairs, in order to prove that our method is indeed ahead of REW, rather than due to statistical bias, we performed a Wilcoxon test using MATLAB 2018b on all scores of our method and REW. The resultant *p*-values of 0.0106 and h = 1 prove that the scores of REW and our method come from different distributions, our method has the better overall performance, and our method can maintain a desirable operation efficiency while guaranteeing the final image quality. Our method can have broad applications and promotion significance.

### 4.2. Compatibility of SC-AOF Method

The SC-AOF method can not only be used independently to generate stitched image with reduced perspective deformation and low alignment error, but also be decomposed (into SC method and image blending method) and combined with other methods to improve the quality of the mosaic.

#### 4.2.1. SC Module Compatibility Analysis

The sliding camera (SC) module in the SC-AOF method can not only be used in the global alignment model, but also be combined with other local alignment models (e.g., APAP and TFT) to solve perspective deformation while maintaining the alignment accuracy. The implementation steps are as follows.

1. Use the global similarity transformation to project $I_{2}$ onto the $I_{1}$ coordinate system to calculate the size and mesh vertices of the mosaic;
2. Use Equations (6)–(9) to calculate the weights of mesh vertices and the projection matrix, replace the homography H in (2) with the homography matrix in local alignment model, and bring them into (12) to compute the warped images and blend them.

**Figure 13:**
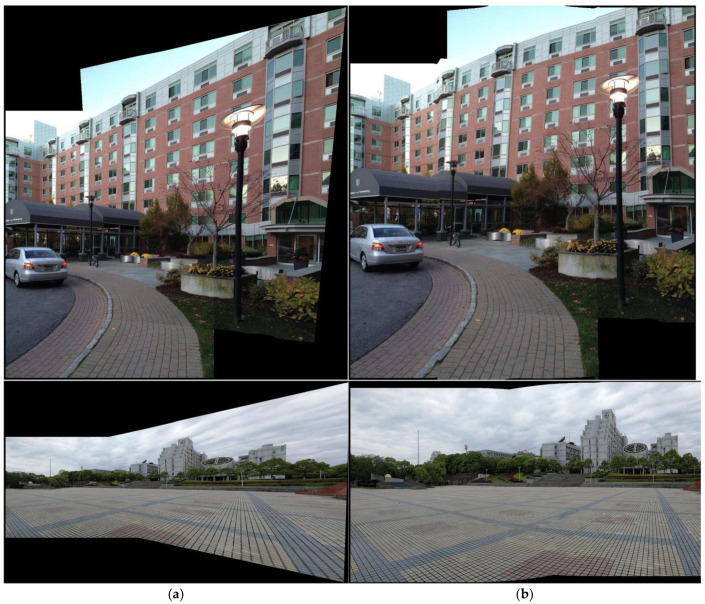
The combination of TFT and moving cameras method. (**a**) The mosaics created using TFT. (**b**) The mosaics obtained by adding the moving camera method to TFT.

Figure 13 presents the stitched images when using the TFT algorithm alone vs. using the TFT algorithm combined with the SC method. The combined method is more effective in mitigating edge stretching, and it generates more natural images. This shows that the SC method can effectively solve perspective deformation suffered by the local alignment method.

#### 4.2.2. Blending Module Compatibility Analysis

The asymmetric optical-flow-based blending in the SC-AOF method can also be used in other methods to enhance the final stitching effect. The implementation steps are as follows.

1. Generate two projected images using one of the other algorithms and calculate the blending parameters based on the overlapping areas;
2. Set the optical flow value to be 0, replace linear blending parameter λ with α in Equation (17) to blend warped images, preserve the blending band width in the low-frequency area and narrow the blending width in the high-frequency area to obtain a better image stitching effect.

**Figure 14:**
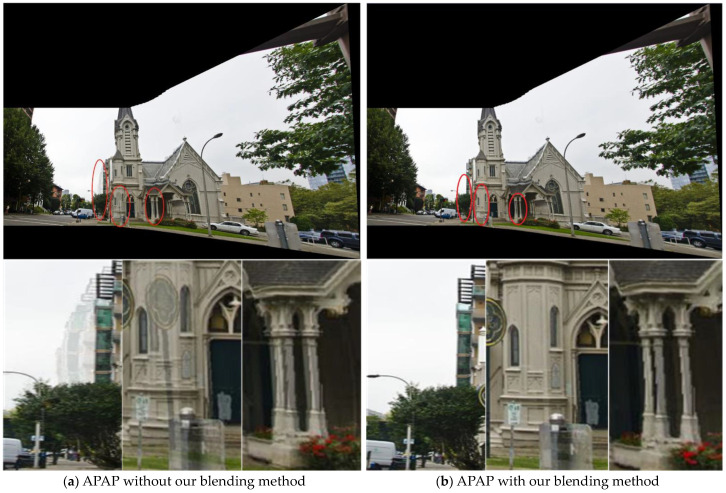
The combination of APAP and our blending method. (**a**) The mosaic and detail view generated by the APAP using linear blending. (**b**) The results of APAP combined with our blending method. The red elliptical boxes indicate the regions where the enlarged images are located.

Figure 14 shows the image stitching effect of the APAP algorithm when using linear blending vs. when using our blending method. It can be seen that the blurring and ghosting in the stitched image are effectively mitigated when using our blending method. This shows that our blending algorithm can blend the aligned images better.

## 5. Conclusions

In this paper, to solve the perspective deformation and misalignment in image stitching using homographies, a SC-AOF method is proposed. In image warping, a new virtual camera and a projection matrix are generated as the observation perspective in the overlapping area by interpolating between two projection matrices. The overlapping area transitions gradually from one viewpoint to another to achieve preservation of the viewpoint and the smooth transition of the stitched image, and thus solve the perspective deformation problem. In image blending, the optical-flow-based blending algorithm is proposed to further improve alignment accuracy. The width of the blending area is automatically adjusted according to the softmax function and alignment accuracy. Finally, extensive comparison experiments are conducted to demonstrate the effectiveness of our algorithm in reducing perspective deformation and improving alignment accuracy. In addition, our algorithm had broad applicability, as its component modules can be used with other algorithms to mitigate edge stretching and improve alignment accuracy.

However, the proposed local alignment method may fail if the input images contain large parallax, which cause severe occlusion to prevent us from obtaining the correct optical flow. The problem of local alignment failure caused by large parallax also exists in other local alignment methods. Exploring more robust optical flow calculation and occlusion processing methods to reduce misalignment in a large parallax scene is an interesting research direction for future work.

## Figures and Tables

**Table 1:** Comparison of SSIM.

	APAP	AANAP	SPHP	TFT	REW	SPW	Ours
**building1**	0.88	0.87	0.75	0.88	0.89	0.86	**0.90**
**building2**	0.82	0.82	0.75	0.92	0.76	0.81	**0.93**
**garden**	0.90	0.92	0.81	0.82	**0.95**	0.92	0.93
**building3**	0.93	0.94	0.89	0.70	**0.96**	0.90	**0.96**
**school**	0.89	0.91	0.67	0.90	0.91	0.87	**0.93**
**wall**	0.83	0.91	0.68	0.90	0.82	0.81	**0.92**
**park-square**	0.95	0.96	0.80	0.97	**0.97**	0.95	**0.97**
**cabinet**	0.91	0.91	0.87	0.89	**0.98**	0.92	0.96
**campus-square**	0.92	0.94	0.84	0.95	**0.98**	0.93	0.97
**racetracks**	0.74	0.79	0.68	**0.86**	0.83	0.70	0.85

**Table 2:** The soring results on the image pairs.

	APAP	AANAP	SPHP	TFT	REW	SPW	Ours
**building1**	1 + 2 + 2 + 2 = 7	2 + 2 + 2 + 0 = 6	2 + 2 + 0 + 2 = 6	1 + 2 + 2 + 1 = 6	**1 + 2 + 2 + 3 = 8**	1 + 2 + 1 + 2 = 6	**2 + 2 + 2 + 2 = 8**
**building2**	0 + 1 + 1 + 2 = 4	1 + 2 + 1 + 0 = 4	2 + 0 + 0 + 2 = 4	1 + 0 + 3 + 0 = 4	1 + 2 + 0 + 3 = 6	1 + 1 + 1 + 2 = 5	**2 + 2 + 3 + 2 = 9**
**garden**	1 + 2 + 2 + 1 = 6	2 + 2 + 2 + 0 = 6	2 + 1 + 0 + 1 = 4	1 + 1 + 0 + 2 = 4	**1 + 2 + 3 + 3 = 9**	2 + 2 + 2 + 1 = 7	**2 + 2 + 3 + 2 = 9**
**building3**	1 + 1 + 2 + 2 = 6	2 + 1 + 2 + 0 = 5	2 + 2 + 1 + 2 = 7	0 + 0 + 0 + 0 = 0	**1 + 2 + 2 + 3 = 8**	1 + 2 + 2 + 2 = 7	**2 + 2 + 2 + 2 = 8**
**school**	0 + 2 + 2 + 2 = 6	2 + 2 + 2 + 0 = 6	2 + 2 + 0 + 2 = 6	0 + 2 + 2 + 2 = 6	0 + 2 + 2 + 2 = 6	1 + 2 + 2 + 2 = 7	**2 + 2 + 2 + 2 = 8**
**wall**	0 + 1 + 1 + 2 = 4	2 + 2 + 2 + 0 = 6	2 + 0 + 0 + 2 = 4	1 + 2 + 2 + 1 = 6	1 + 1 + 1 + 2 = 5	0 + 1 + 1 + 2 = 4	**2 + 2 + 3 + 2 = 9**
**park-square**	1 + 2 + 2 + 2 = 7	1 + 2 + 2 + 0 = 5	2 + 0 + 0 + 1 = 3	1 + 2 + 2 + 2 = 7	**1 + 2 + 2 + 3 = 8**	1 + 2 + 2 + 0 = 5	**2 + 2 + 2 + 2 = 8**
**cabinet**	1 + 1 + 1 + 2 = 5	2 + 1 + 1 + 0 = 4	2 + 2 + 0 + 2 = 6	2 + 0 + 1 + 2 = 5	**2 + 2 + 3 + 2 = 9**	1 + 2 + 2 + 2 = 7	**2 + 2 + 3 + 2 = 9**
**campus-square**	0 + 2 + 1 + 2 = 5	1 + 2 + 2 + 0 = 5	2 + 2 + 0 + 2 = 6	0 + 2 + 2 + 2 = 6	**0 + 2 + 3 + 3 = 8**	0 + 2 + 1 + 2 = 5	**2 + 2 + 2 + 2 = 8**
**racetracks**	2 + 1 + 1 + 2 = 6	2 + 1 + 2 + 0 = 5	2 + 0 + 0 + 1 = 3	**2 + 2 + 3 + 2 = 9**	**2 + 2 + 2 + 3 = 9**	1 + 1 + 0 + 1 = 3	**2 + 2 + 3 + 2 = 9**
**roundabout**	**2 + 2 + 2 + 2 = 8**	2 + 2 + 2 + 0 = 6	2 + 2 + 0 + 1 = 5	2 + 1 + 2 + 1 = 6	**2 + 2 + 2 + 2 = 8**	2 + 2 + 0 + 2 = 6	**2 + 2 + 2 + 2 = 8**
**fence**	1 + 2 + 2 + 2 = 7	2 + 2 + 2 + 0 = 6	2 + 1 + 0 + 2 = 5	1 + 1 + 2 + 1 = 5	1 + 2 + 2 + 2 = 7	**2 + 2 + 2 + 2 = 8**	**2 + 2 + 2 + 2 = 8**
**railtracks**	2 + 1 + 1 + 2 = 6	2 + 2 + 2 + 0 = 6	2 + 0 + 0 + 2 = 4	2 + 2 + 2 + 2 = 8	2 + 2 + 2 + 2 = 8	2 + 1 + 1 + 0 = 4	**2 + 2 + 3 + 2 = 9**
**temple**	1 + 2 + 2 + 2 = 7	2 + 2 + 2 + 0 = 6	2 + 2 + 0 + 2 = 6	1 + 1 + 2 + 2 = 4	1 + 2 + 2 + 2 = 7	1 + 1 + 1 + 2 = 5	**2 + 2 + 2 + 2 = 8**
**corner**	2 + 2 + 2 + 1 = 7	2 + 1 + 2 + 0 = 5	2 + 1 + 1 + 2 = 6	2 + 0 + 0 + 2 = 4	**2 + 2 + 2 + 2 = 8**	2 + 1 + 2 + 2 = 7	**2 + 2 + 2 + 2 = 8**
**shelf**	2 + 2 + 2 + 1 = 7	2 + 2 + 2 + 0 = 6	2 + 2 + 0 + 2 = 6	2 + 1 + 2 + 2 = 7	**2 + 2 + 2 + 3 = 9**	2 + 2 + 2 + 1 = 7	2 + 2 + 2 + 2 = 8
**standing-he**	1 + 1 + 2 + 2 = 6	2 + 1 + 1 + 1 = 5	2 + 1 + 1 + 2 = 6	0 + 0 + 1 + 2 = 3	1 + 1 + 2 + 2 = 6	1 + 1 + 2 + 0 = 4	**2 + 1 + 3 + 2 = 8**
**foundation**	1 + 2 + 2 + 2 = 7	2 + 2 + 2 + 0 = 6	2 + 2 + 0 + 2 = 6	1 + 1 + 3 + 2 = 7	**2 + 2 + 2 + 2 = 8**	1 + 2 + 1 + 0 = 4	**2 + 2 + 2 + 2 = 8**
**guardbar**	1 + 1 + 2 + 1 = 5	2 + 1 + 2 + 0 = 5	2 + 1 + 0 + 2 = 5	1 + 1 + 3 + 2 = 7	1 + 1 + 2 + 2 = 6	1 + 1 + 1 + 1 = 4	**2 + 1 + 2 + 2 = 7**
**office**	1 + 1 + 2 + 2 = 6	2 + 1 + 2 + 0 = 5	2 + 1 + 0 + 2 = 5	0 + 1 + 2 + 1 = 4	1 + 1 + 1 + 3 = 6	0 + 1 + 2 + 1 = 4	**2 + 2 + 3 + 2 = 9**
**plantain**	1 + 2 + 2 + 2 = 7	1 + 2 + 2 + 0 = 5	2 + 2 + 1 + 2 = 7	0 + 0 + 0 + 1 = 1	**2 + 1 + 2 + 3 = 8**	1 + 2 + 2 + 2 = 7	**2 + 2 + 2 + 2 = 8**
**building4**	1 + 1 + 2 + 1 = 5	2 + 1 + 2 + 0 = 5	2 + 1 + 0 + 2 = 5	1 + 1 + 2 + 2 = 6	1 + 2 + 2 + 2 = 7	1 + 2 + 1 + 2 = 6	**2 + 2 + 3 + 2 = 9**
**potberry**	**2 + 2 + 2 + 2 = 8**	**2 + 2 + 2 + 2 = 8**	2 + 2 + 0 + 2 = 6	2 + 2 + 2 + 1 = 7	**2 + 1 + 2 + 3 = 8**	1 + 1 + 1 + 0 = 3	**2 + 2 + 2 + 2 = 8**
**lawn**	1 + 2 + 2 + 2 = 7	1 + 2 + 2 + 2 = 7	2 + 2 + 0 + 0 = 4	1 + 2 + 2 + 1 = 6	**1 + 2 + 2 + 3 = 8**	1 + 2 + 2 + 1 = 6	**2 + 2 + 2 + 2 = 8**
**worktable**	2 + 1 + 2 + 2 = 7	2 + 1 + 1 + 0 = 4	1 + 1 + 0 + 2 = 4	2 + 0 + 2 + 2 = 6	**2 + 2 + 2 + 2 = 8**	2 + 1 + 2 + 0 = 5	**2 + 2 + 2 + 2 = 8**

**Figure A1:**
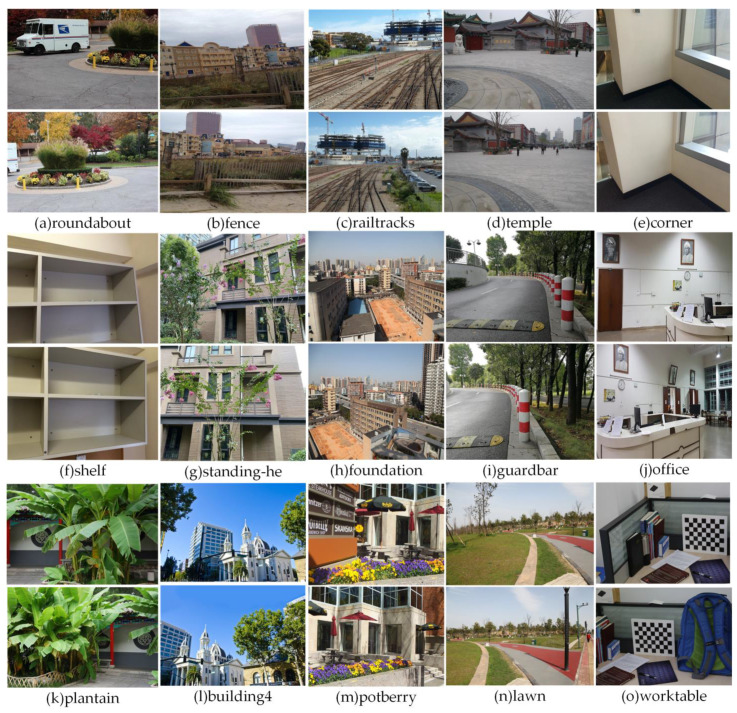
The image dataset for supplementary comparative experiments. The image pairs are initially used by stitching methods such as APAP, AANAP, REW. Four yellow Chinese artistic characters are shown on the temple’s wall in the upper image of (**d**).

**Figure A2:**
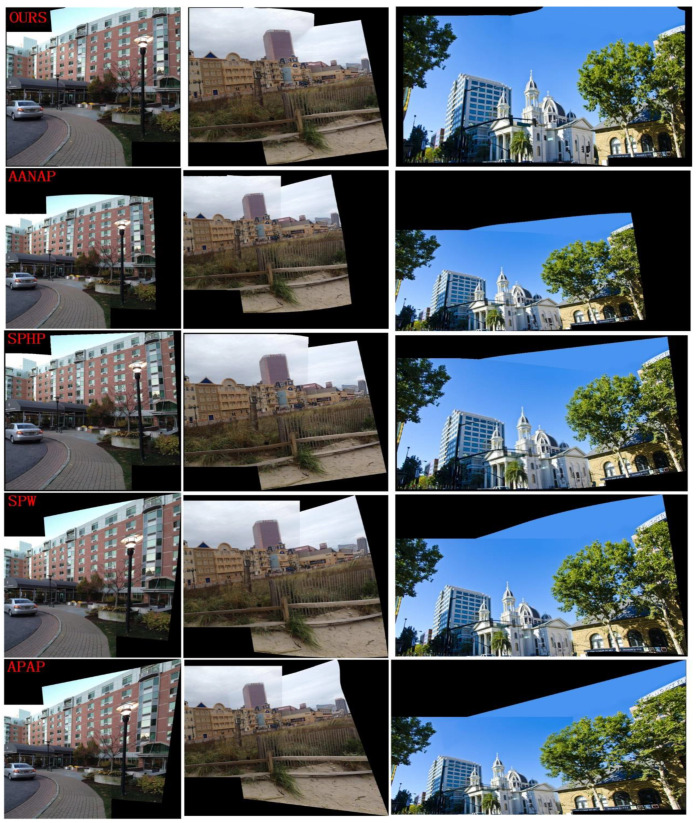
Qualitative comparisons of perspective deformation reduction on the building1, fence and building4 image pairs.

**Figure A3:**
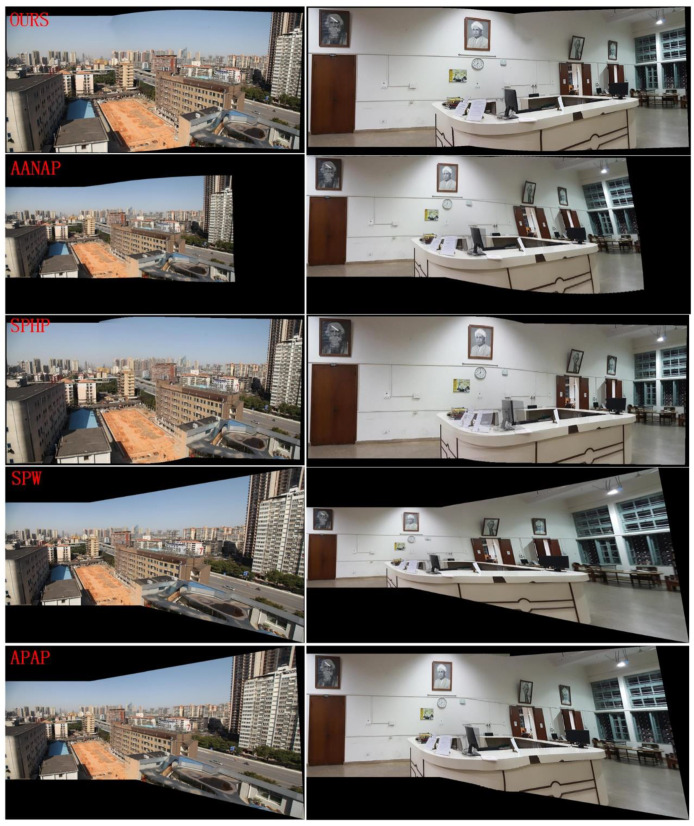
Qualitative comparisons of perspective deformation reduction on the foundation and office image pairs.

**Figure A4:**
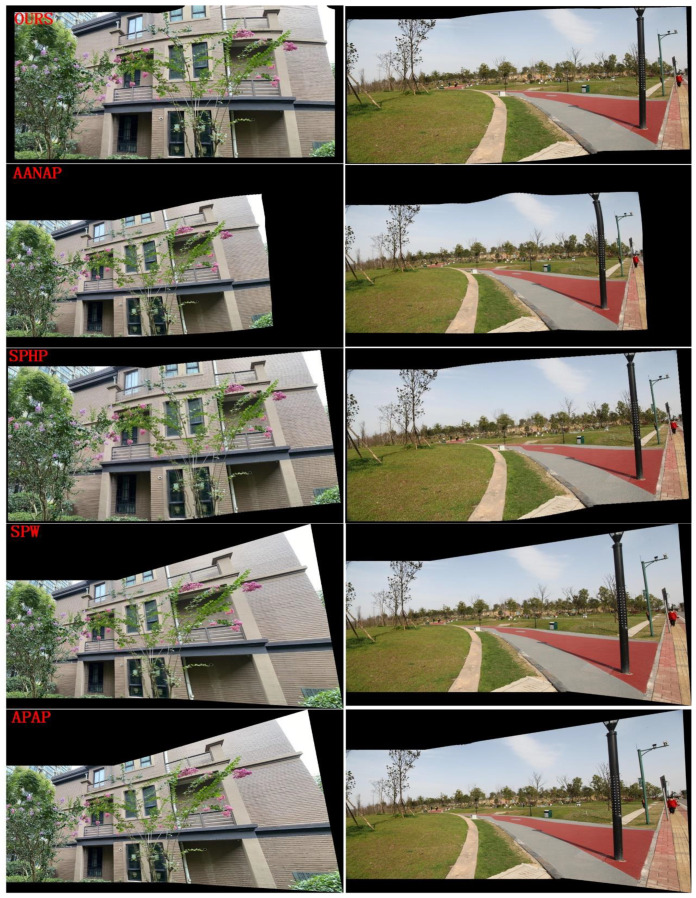
Qualitative comparisons of perspective deformation reduction on the standing-he and lawn image pairs.

**Figure A5:**
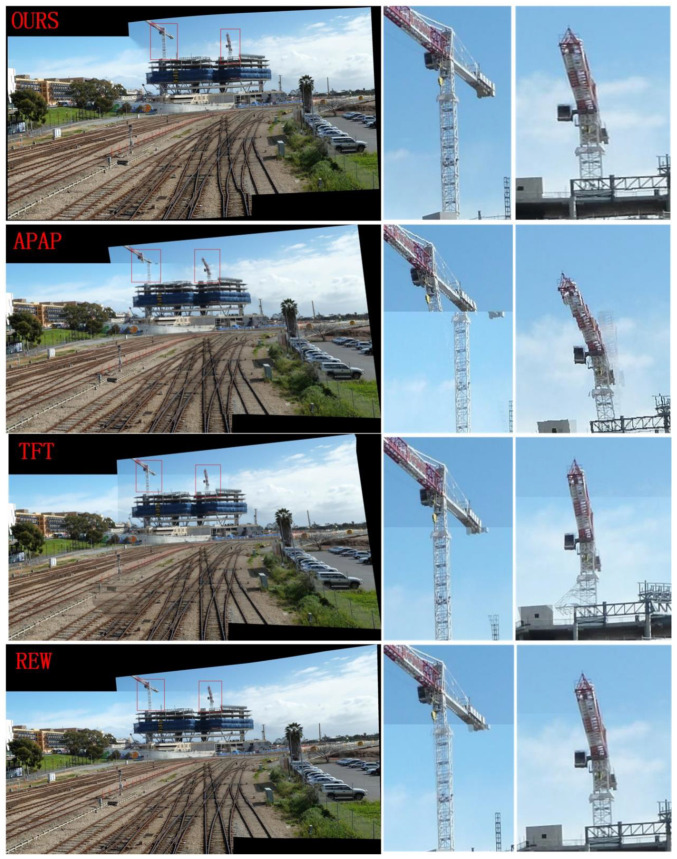
Qualitative comparisons of local alignment on the railtracks image pair. The red boxes indicate the regions where the enlarged images are located in the mosaics.

**Figure A6:**
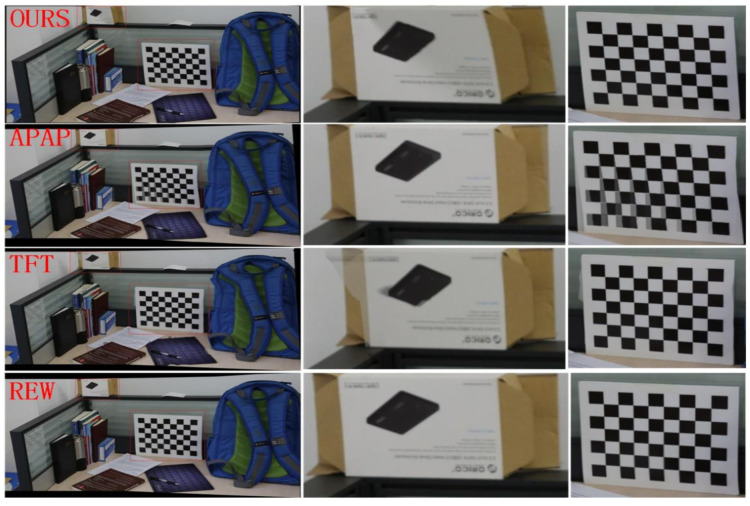
Qualitative comparisons of local alignment on the worktable image pair. The red boxes indicate the regions where the enlarged images are located in the mosaics.

**Figure A7:**
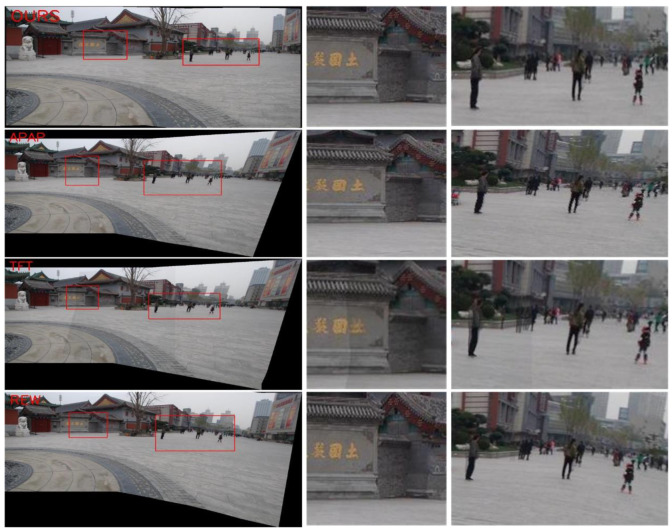
Qualitative comparisons of local alignment on the temple image pair. The red boxes indicate the regions where the enlarged images are located in the mosaics.

**Figure A8:**
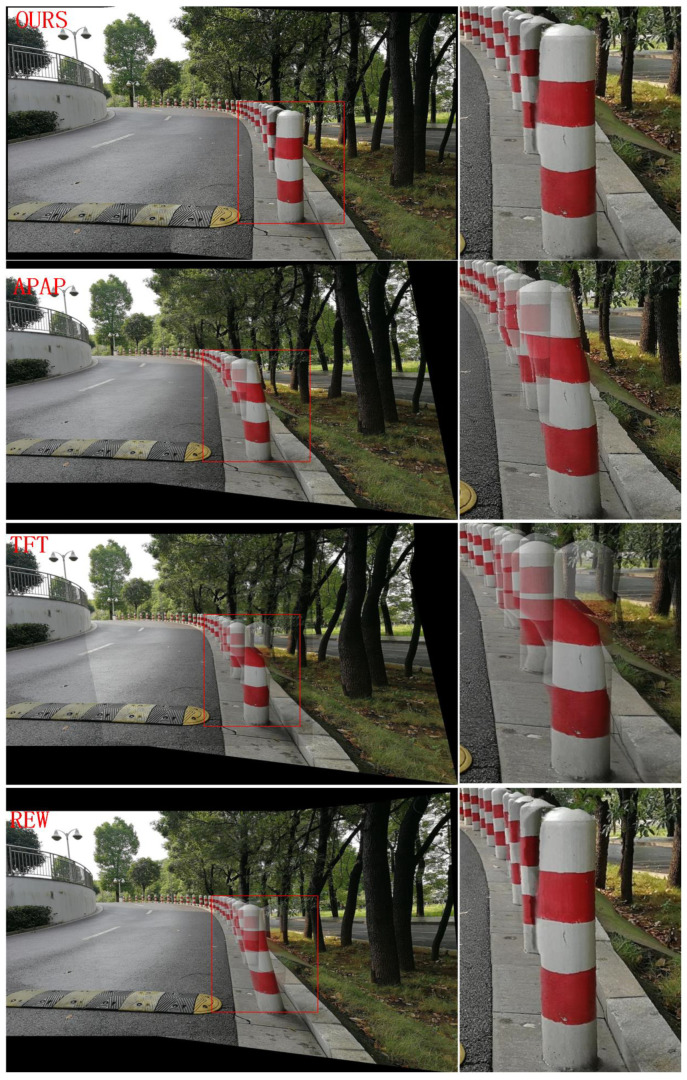
Qualitative comparisons of local alignment on the guardbar image pair. The red boxes indicate the regions where the enlarged images are located in the mosaics.

**Figure A9:**
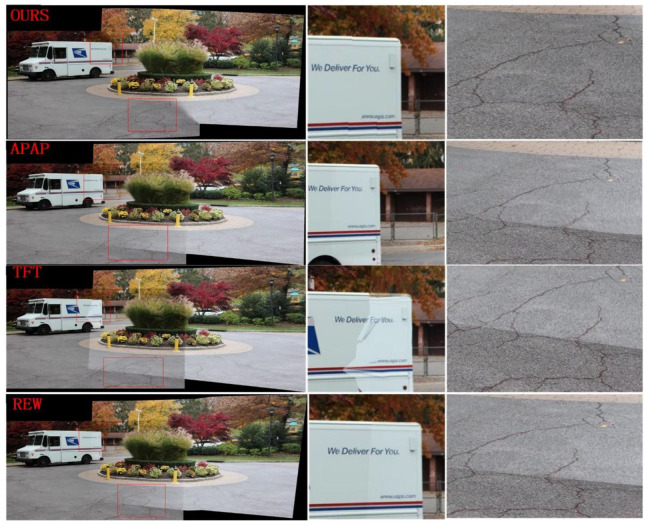
Qualitative comparisons of local alignment on the roundabout image pair. The red boxes indicate the regions where the enlarged images are located in the mosaics.

**Figure A10:**
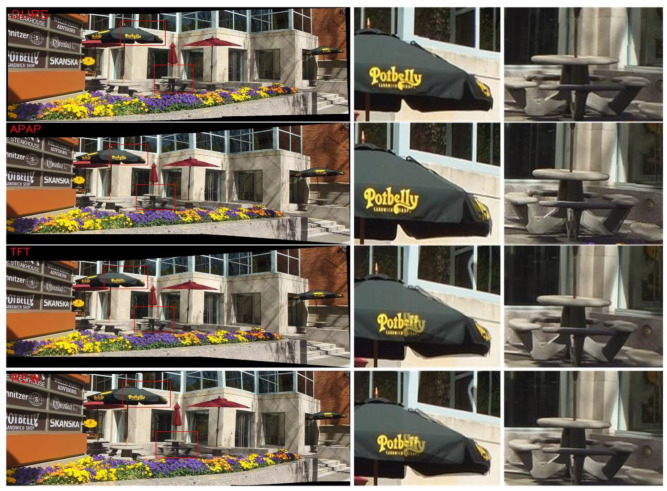
Qualitative comparisons of local alignment on the potberry image pair. The red boxes indicate the regions where the enlarged images are located in the mosaics.

**Figure A11:**
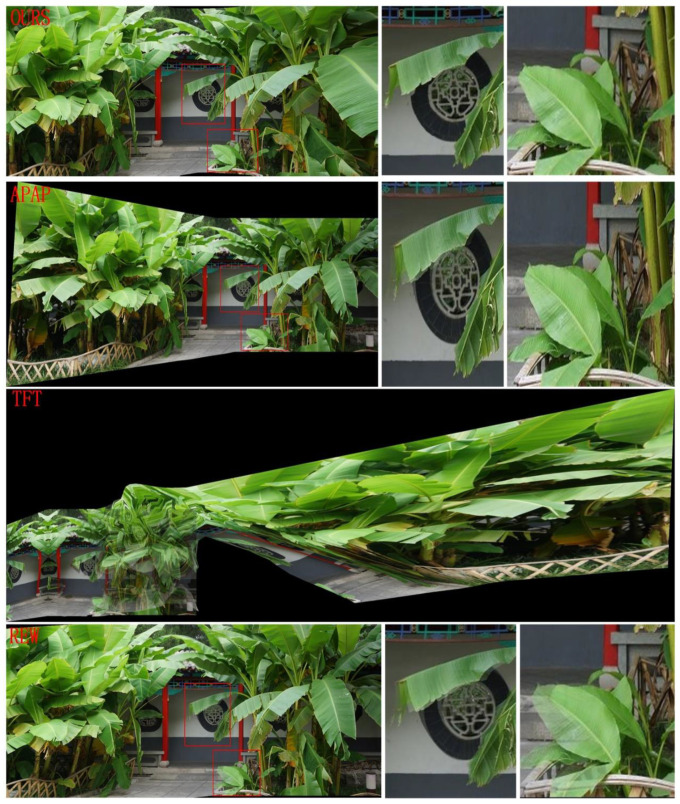
Qualitative comparisons of local alignment on the plantain image pair. TFT failed to stitch this image pair. The red boxes indicate the regions where the enlarged images are located in the mosaics.

**Figure A12:**
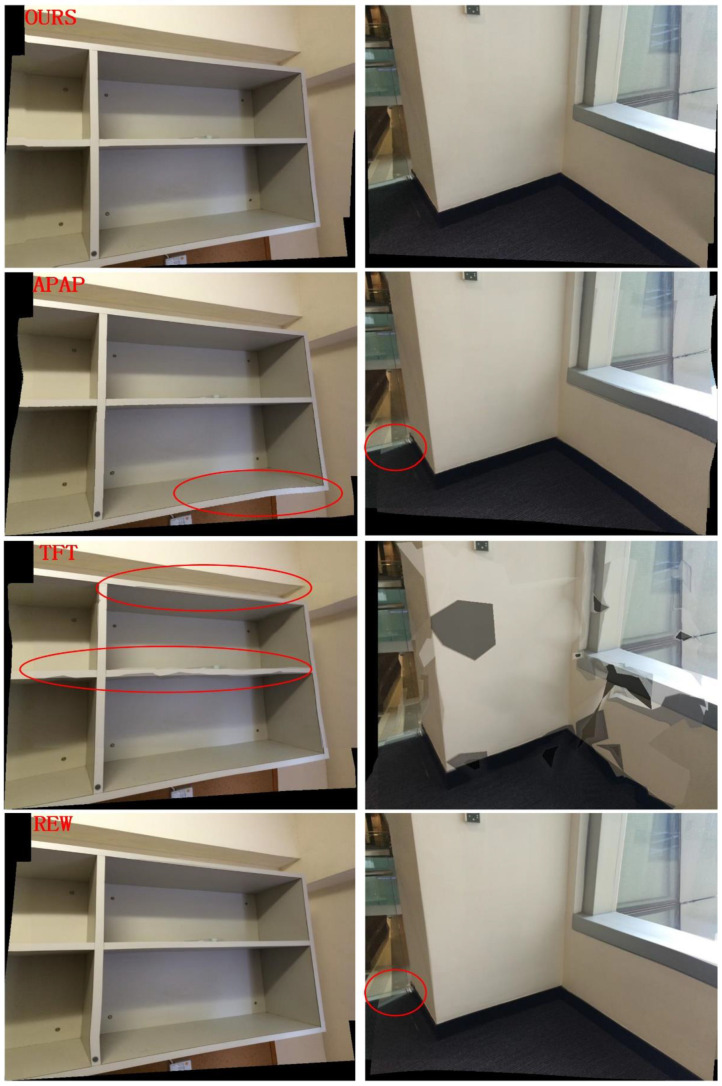
Qualitative comparisons of local alignment on the shelf and corner image pairs. TFT failed to stitch the corner image pair. The Red circles highlight errors and distortions.

**Figure A13:**
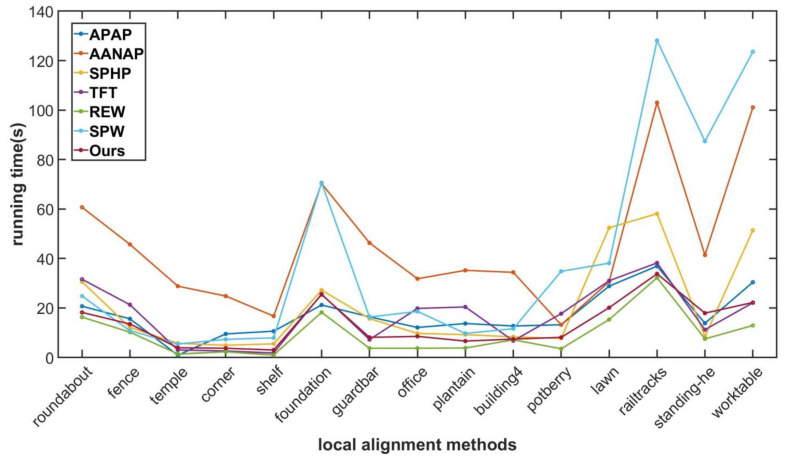
Comparison on elapsed time. Our method is only second to REW in speed and is superior to other methods.

## Data Availability

The raw data supporting the conclusions of this article will be made available by the authors on request.

## Appendix A

Given that the abundance of symbols and abbreviations in this paper can lead to reading confusion, the symbols and abbreviations are listed and explained in Table A1 and Table A2.

**Table A1:** The symbols and their explanations.

Symbols	Description
$I_{1},I_{2}$,I	the source image pair and the final mosaic
n	the global projection plane
$\Omega_{1},\Omega_{2}$	the non-overlapping area of $I_{1},I_{2}$
$\Omega_{0}$	the overlapping area of $I_{1}$ and $I_{2}$
$P_{1},P_{2}$	the camera projection matrix of $I_{1},I_{2}$
$P_{im},P_{m}$	the projection matrix of virtual sliding camera
$P_{2}^{\prime }$	the adjusted projection matrix of $I_{2}$ to unify the pixel coordinates of $I_{1}$ and $I_{2}$
$u^{i},u$	the non-homogeneous coordinate of the pixel in I
${\tilde{u}}^{i},\tilde{u}$	the homogeneous coordinate of the pixel in I
$u_{1}^{i},u_{1},u_{2}^{i},u_{2}$	the non-homogeneous coordinate of the pixel in $I_{1},I_{2}$
${\tilde{u}}_{1}^{i},{\tilde{u}}_{1},{\tilde{u}}_{2}^{i},{\tilde{u}}_{2}$	the homogeneous coordinate of the pixel in $I_{1},I_{2}$
${S,S}_{i}$	the sampling points on the plane n which are projected onto $u\;and\;u^{i}$ in I
$p,p_{i}$	the 3D scene points
${C_{1},C}_{2}$	the cameras corresponding to $I_{1}$ and $I_{2}$
$K_{1},K_{2}$	the internal parameter matrices of ${C_{1}\;and\;C}_{2}$
$I_{3\times 3}$	the 3 by 3 identity matrix
R,t	the rotation matrix and the location of the optical center of $C_{2}$ in $C_{1}\prime s\;$ coordinate system
${\tilde{y}}_{1},{\tilde{y}}_{2}$	normalized coordinates of ${\tilde{u}}_{1}$ and ${\tilde{u}}_{2}$
$N_{*}$	the similarity matrix between $I_{1}$ and $I_{2}$
$K_{2}^{\prime },R_{2}^{\prime }$	the rotation matrix and translation vector corresponding to $P_{2}^{\prime }$
$K_{m},R_{m},t_{m}$	the internal parameter matrix, the rotation matrix and the translation vector corresponding to $P_{m}$
$q_{1},q_{2},q_{m}$	the quaternions corresponding to $I_{3\times 3}$, $R_{2}^{\prime }$ and $R_{m}$
$H_{m}^{i}\left(i=1,2\right)$	the homography between $I_{i}$ and I
$I_{i}^{\prime }\left(i=1,2\right)$	the warped image of $I_{i}$ using $H_{m}^{i}$
$F_{i\to j}\left(p_{i}\right)$	the optical flow of $p_{i}$ in $I_{i}^{\prime }$ which makes $I_{i}^{\prime }(p_{i})=I_{j}^{\prime }(p_{i}+F_{i\to j}\left(p_{i}\right))$
$\tilde{p},{\tilde{p}}_{1},{\tilde{p}}_{2}$	the pixel in I and corresponding pixel in $I_{1}^{\prime },I_{2}^{\prime }$
$M_{1},M_{2}$	the optical flow magnitude of $F_{2\to 1}\left(\tilde{p}\right)$ and $F_{1\to 2}\left(\tilde{p}\right)$
$\alpha_{s},\alpha_{m}$	the softmax function’s shape coefficien and the optical flow’s enhancement coefficient
λ	the weight which makes I transition from $I_{1}^{\prime }$ to $I_{2}^{\prime }$ linearly
β	the softmax weight to transition I from $I_{1}^{\prime }$ to $I_{2}^{\prime }$ fastly
α	the linear combinationof λ and β which makes the transition of I from $I_{1}^{\prime }$ to $I_{2}^{\prime }$ depends on the color difference
$D_{c},\lambda_{d}$	the color difference and the hyperbolic tangent function of the color difference
F(u)	the optical flow of u
E(F(u))	the error function of F(u) used for solving the optimal optical flow
$E_{I},E_{S},E_{T}$	the optical flow’s alignment error, consistency error and penalty for large value
$F_{inlier}^{i},H^{i}$	the i-th homography transforming $I_{1}$ to $I_{2}$ and the corresponding inlier set
$F_{cond},F_{inlier}$	the initial set and the final inlier set of matched feature points
$r_{e},r_{p}$	the projection errors of the epipolar constraint and of the infinite homography constraint
h(r,σ)	the robust kernel function to reduce the impact of false matches to optimization

**Table A2:** The abbreviations and their explanations.

Abbreviation	Meaning
SC	sliding camera, proposed by us to solve the perspective deformation
AOF	asymmetric optical flow, proposed by us to slove the local alignment
APAP	as-projective-as-possible, used to solve the local alignment by location-dependent homography warping
DLT	direct linear transform, used for estimating the parameters of the homography
REW	robust elastic warping, used to improve the local alignment using deformation fields
TPS	thin-plate spline, used to compute deformation fields corresponding to matched feature points
TFT	triangular facet approximation, using scene triangular facet estimating to improve the local alignment
NIS	natural image stitching, a local alignment method using the depth map
SPHP	shape preserving half projective, solving perspective deformation by gradually changing the resultant warp from projective to similarity
AANAP	adaptive as-natural-as-possible, a method to solve perspective deformation
GSP	global similarity prior, used to align images and reduce deformation
SPW	single-projective warp, which adopts the quasi-homography warp to mitigate projective distortion and preserve single perspective
SPSO	structure preservation and seam optimization, a method can obtain precise alignment while preserving local and global image structures.
GES-GSP	geometric structure preserving-global similarity prior, based on GSP to futher protect the large-scale geometric structure from distortion
SIFT	scale-invariant feature transform, a feature detection and description method
SURF	speed-up robust feature, a feature detection and description method, faster than SIFT
KNN	k-nearest neighbor, a feature matching method
RAFT	recurrent all-pairs field transforms, estimating optical flow based on deep learning
RANSAC	random sample consensus, used to filter outliers and estimate model parameters

## Appendix B

In this section, some supplementary experiments about perspective deformation reduction and local alignment are added. The image pairs used in the supplementary experiments are shown in Figure A1.

Figure A2, Figure A3 and Figure A4 show the comparisons of perspective deformation reduction of our method, AANAP, SPHP, SPW and APAP.

The comparisons of local alignment of our method, APAP, TFT and REW are shown in Figure A5, Figure A6, Figure A7, Figure A8, Figure A9, Figure A10, Figure A11 and Figure A12. The detail images inside the red rects are the image regions with misalignment and shown directly right the mosaics.

The scores of all methods on the image pairs roundabout, fence, railtracks, temple, corner, shelf, standing-he, foundation, guardbar, office, plantain, building4, potberry, lawn and worktable are listed in Table A3. The best SSIM value is highlighted in bold.

**Table A3:** Comparison on SSIM.

	APAP	AANAP	SPHP	TFT	REW	SPW	Ours
**roundabout**	0.85	0.86	0.77	0.86	0.86	0.76	**0.87**
**fence**	0.93	**0.95**	0.81	**0.95**	**0.95**	0.93	**0.95**
**railtracks**	0.77	0.90	0.62	0.92	0.85	0.77	**0.94**
**temple**	0.90	0.91	0.73	0.95	0.94	0.85	**0.96**
**corner**	**0.98**	0.97	0.91	0.73	**0.98**	0.97	0.96
**shelf**	**0.98**	**0.98**	0.84	0.95	0.97	0.96	0.97
**standing-he**	0.72	0.77	0.64	0.35	0.78	0.71	**0.84**
**foundation**	0.75	0.78	0.58	**0.83**	0.76	0.71	0.80
**guardbar**	0.74	0.74	0.58	**0.79**	0.77	0.65	0.76
**office**	0.79	0.78	0.55	0.84	0.65	0.75	**0.88**
**plantain**	0.85	0.85	0.67	0.31	0.82	0.85	**0.90**
**building4**	0.71	0.72	0.58	0.73	0.74	0.70	**0.78**
**potberry**	0.89	0.89	0.68	**0.93**	0.87	0.82	0.91
**lawn**	0.92	**0.95**	0.79	**0.95**	**0.95**	0.93	**0.95**
**worktable**	0.87	0.84	0.59	0.86	**0.97**	0.85	**0.97**

Figure A13 shows the speed of the SC-AOF method versus the APAP, AANAP, SPHP, TFT, REW and SPW methods. The same image pairs as in the SSIM comparison are used in this experiment.
